# Synthesis and Evaluation of Ciprofloxacin-Nitroxide Conjugates as Anti-Biofilm Agents

**DOI:** 10.3390/molecules21070841

**Published:** 2016-06-27

**Authors:** Anthony D. Verderosa, Sarah C. Mansour, César de la Fuente-Núñez, Robert E. W. Hancock, Kathryn E. Fairfull-Smith

**Affiliations:** 1 ARC Centre of Excellence for Free Radical Chemistry and Biotechnology, Faculty of Science and Engineering, Queensland University of Technology, Queensland 4001, Australia; a.verderosa@hdr.qut.edu.au; 2 Centre for Microbial Diseases and Immunity Research, Department of Microbiology and Immunology, University of British Columbia, Vancouver, BC V6T 1Z4, Canada; sarah@hancocklab.com (S.C.M.); bob@hancocklab.com (R.E.W.H.); 3 Synthetic Biology Group, MIT Synthetic Biology Center, Massachusetts Institute of Technology, Cambridge, MA 02139, USA; cfuente@mit.edu; 4 Research Laboratory of Electronics, Department of Biological Engineering, Department of Electrical Engineering and Computer Science, Massachusetts Institute of Technology, Cambridge 02139, MA, USA; 5 Broad Institute of MIT and Harvard, Cambridge, MA 02142, USA; 6 Harvard Biophysics Program, Harvard University, Boston, MA 02115, USA

**Keywords:** radical, antibiotic, biofilm, nitroxide, ciprofloxacin

## Abstract

As bacterial biofilms are often refractory to conventional antimicrobials, the need for alternative and/or novel strategies for the treatment of biofilm related infections has become of paramount importance. Herein, we report the synthesis of novel hybrid molecules comprised of two different hindered nitroxides linked to the piperazinyl secondary amine of ciprofloxacin via a tertiary amine linker achieved utilising reductive amination. The corresponding methoxyamine derivatives were prepared alongside their radical-containing counterparts as controls. Subsequent biological evaluation of the hybrid compounds on preformed *P. aeruginosa* flow cell biofilms divulged significant dispersal and eradication abilities for ciprofloxacin-nitroxide hybrid compound **10** (up to 95% eradication of mature biofilms at 40 μM). Importantly, these hybrids represent the first dual-action antimicrobial-nitroxide agents, which harness the dispersal properties of the nitroxide moiety to circumvent the well-known resistance of biofilms to treatment with antimicrobial agents.

## 1. Introduction

The adhesion of planktonic bacterial cells to a surface in the presence of the appropriate environmental cues often results in the formation of a complex bacterial community known as a biofilm. Biofilms represent a significant problem for almost all healthcare systems around the world [1,2,3], mainly due to their ability to colonize indwelling medical devices [4] and chronic wounds [5]. A large proportion (approximately 80%) of all microbial-based infections present in humans [6] are caused by biofilms. Although numerous antimicrobial treatments exist for the effective eradication of planktonic bacteria, these approaches are generally ineffective when implemented against biofilms [7,8], which have been shown to be extremely resistant to antibiotic therapies [6,9,10]. Consequently, the development of novel strategies for the effective treatment of mature biofilms is urgently required. 

Although the natural mode of bacterial growth is well known to be predominately as a biofilm, it is also understood that the bacterial population can alternate between the planktonic and biofilm lifestyles by varying the expression of particular genes [11]. Hence, an emerging strategy to combat bacterial biofilms is to use small molecules that act through non-microbicidal mechanisms to inhibit and/or disperse bacterial biofilms [12,13]. The free radical gaseous molecule, nitric oxide (NO), has been identified as an important signaling molecule in a variety of biofilm-forming species [14] as it is capable of mediating both biofilm formation and dispersal [15,16,17]. Nitric oxide can induce a transition from the sessile biofilm mode of growth to a dispersed (planktonic) state when utilised at low, sub-lethal concentrations (in the pM to low nM range) [14,16]. Interestingly, differences in the phenotypes of dispersed and planktonic cells have been recently reported in *P. aeruginosa* [18]. The observed effect of NO on biofilms has been associated with a reduction in the intracellular levels of the secondary messenger cyclic di-GMP, which plays a key role in biofilm development [11,19]. 

**Figure 1:**
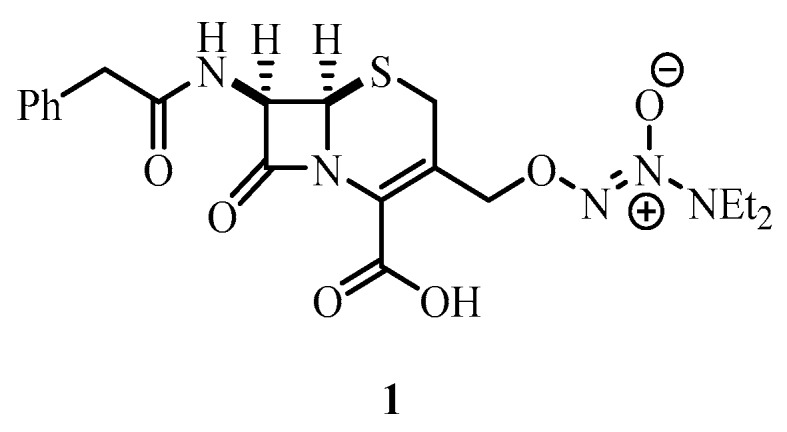
NO donor prodrug cephalosporin-3′-diazeniumdiolate **1**.

The implementation of nitric oxide as an anti-biofilm agent is significantly hindered by its extreme chemical reactivity and short half-life (between 0.1–5 s) [20]. Thus, as a means to avoid the challenges associated with handling and/or delivering nitric oxide into systems where biofilms are prevalent, the use of nitric oxide-donor molecules [21] have been investigated and comprehensive reviews on the ability of NO-donor compounds to disperse bacterial biofilms have been recently documented [22]. Several new anti-biofilm compounds have also been developed, which incorporate NO donors such as cephalosporin-3′-diazeniumdiolate **1** (Figure 1) that only releases NO upon reaction with biofilm specific enzymes (e.g., β-lactamase) [23]. However, as NO-donor molecules are also often inherently unstable [24], the use of nitroxides, have more recently been examined as an alternative strategy for biofilm dispersal.

**Figure 2:**
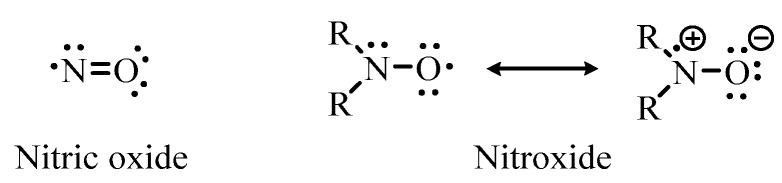
Structure of nitric oxide and the general structure of a nitroxide.

Nitroxides are long-lived, stable free radical species that contain a disubstituted nitrogen atom bound to a univalent oxygen atom [25]. Nitroxides and nitric oxide are structurally similar compounds with both containing an unpaired electron, which is delocalized over the nitrogen-oxygen bond (Figure 2). Additionally, as both types of compounds are known to be efficient scavengers of protein-derived radicals, the biological effects of nitroxides can be explained by their nitric oxide-mimetic properties [26]. However, unlike nitric oxide, which at room temperature is a reactive gas, and relatively unstable, nitroxides are generally air-stable crystalline solids. Nitroxides can also undergo redox chemistry and it has been previously hypothesized that their antibacterial activity may result from their oxidation by reactive oxygen species to oxoammonium ions which interact with bacterial cell membranes [27].

We have previously established that nitroxide-containing compounds can mimic the dispersal behavior of nitric oxide towards *Pseudomonas aeruginosa* biofilms grown in flow cell chambers [27]. At concentrations of 20 µM, nitroxides were shown to both inhibit *P. aeruginosa* biofilm formation, and trigger the dispersal of mature biofilms. Furthermore, these compounds were able to restore swarming motility in a nitrite reductase deficient mutant strain of *P.* *aeruginosa*, indicating that they were acting in a similar manner to nitric oxide and were able to enter bacterial cells. The effect of various nitroxides on biofilm dispersal has also been reported by others but at higher concentrations (5 mM) using the less-sensitive crystal violet staining technique [28,29]. The anti-bacterial activity of nitroxide-coated silver nanoparticles has also been recently described, with the presence of the nitroxide unit shown to enhance the anti-bacterial properties of the silver nanoparticles [30]. Additionally, we have recently demonstrated that almost complete eradication of mature *P. aeruginosa* and *E. coli* biofilms can be achieved when biofilms are treated with a nitroxide in combination with an antibiotic (ciprofloxacin) [31]. These results suggest that the dispersal properties of nitroxides can be harnessed to circumvent the well-known resistance of biofilms to treatment with antimicrobial agents. 

In the present study, we explored the possibility that a nitroxide and an antibiotic could be combined into a single molecule for the efficient treatment of biofilms. The rationale behind this approach was that the nitroxide would trigger the dispersal of cells from biofilms (that are resistant to antibiotic action), and the antibiotic would then be able to kill these dispersed bacteria, thus efficiently eradicating the biofilm. The use of a conjugate molecule in this strategy should optimize the anti-biofilm effect as the antibiotic would be located near the site of biofilm dispersal allowing it to act directly on the dispersed cell population before the bacteria returns to the preferred biofilm mode of growth.

Herein, we report the design and synthesis of several ciprofloxacin-nitroxide hybrid molecules and their evaluation as potential anti-biofilm agents to eradicate existing *P. aeruginosa* biofilms.

## 2. Results and Discussion

### 2.1. Chemistry

**Figure 3:**
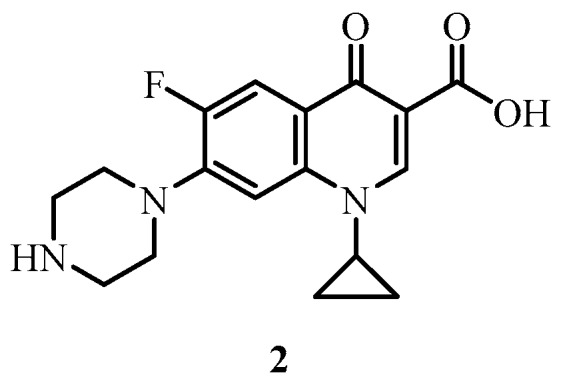
Ciprofloxacin **2**.

Ciprofloxacin **2** (Figure 3) is a fluoroquinolone antibiotic that acts by inhibiting the bacterial enzymes DNA gyrase (a type II topoisomerase) and topoisomerase IV, which are required for DNA replication [32,33]. Various structural modifications to the ciprofloxacin core have disclosed that the secondary amine of the piperazine ring at the 7-position represents a useful handle where further synthetic transformations can be performed without significantly affecting the antimicrobial activity of the molecule [34,35,36]. Furthermore, the addition of large substituents at the 7-position of fluoroquinolones does not adversely affect drug permeability through bacterial membranes [37] and the variety of piperazinyl substituents introduced at this position has revealed the looseness of the binding pocket of the targeted DNA gyrase [38,39,40].

For these reasons, we chose to generate our first generation of ciprofloxacin-nitroxide hybrids by tethering nitroxides to the secondary amine of the piperazine ring of ciprofloxacin, using reductive amination chemistry [41] to generate a ciprofloxacin-nitroxide conjugate connected via a tertiary amine linkage. The cyclic nitroxides 4-oxo-2,2,6,6-tetramethylpiperidin-1-yloxyl (TEMPONE) **6** and the more rigid 5-formyl-1,1,3,3-tetramethylisoindolin-2-yloxyl (FTMIO) **12** [42] were selected as the nitroxide coupling partners as both these nitroxide structures contain bis(*tert*-alkyl) groups on the carbon atoms α to the nitroxide, making them highly resistant to degradation through disproportionation [43]. In addition to this, both of the structural piperidine and isoindoline cores have been previously shown to possess dispersal activities in bacterial biofilms [27].

The reductive amination methodology was first optimized using TEMPONE **6**. To begin, the carboxylic acid of ciprofloxacin **2** was protected by conversion to the ethyl ester derivative **5**, by following previously documented methodologies [44]. Using typical reductive amination conditions [45], a methanol solution of the protected ciprofloxacin **5** with TEMPONE **6** and acetic acid (4 equivalents) was treated with NaBH**_3_**CN. Acetic acid is often used for reductive amination reactions with ketones to catalyze the formation of the imine intermediate [46]. NaBH_3_CN was employed as the reductant as it can selectively reduce imines in the presence of carbonyl groups [47].

**Figure 4:**
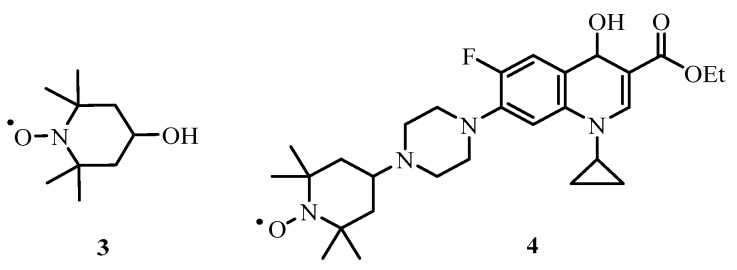
TEMPOL **3** and alcohol derivative **4**.

After heating at 60 °C for 5 h, no starting material remained (TLC analysis) and the desired protected ciprofloxacin-nitroxide hybrid **8** could be isolated but only in a low yield (3%). Two additional products, the alcohol derivative **4** (Figure 4) of **8** and 4-hydroxy-2,2,6,6-tetramethylpiperidin-1-yloxyl (TEMPOL) **3** (Figure 4) were also formed in the reaction and isolated in modest yields (23% and 30% yield).

The reduction of the ketone at the 4-position of ciprofloxacin **2** by NaBH_3_CN has not been previously reported, but the reduction of ketones or aldehydes, to their corresponding alcohols, in the presence of NaBH_3_CN is well documented and reported to be highly pH dependent [48]. In acidic environments (pH < 4), the reduction of ketones or aldehydes with NaBH_3_CN proceeds rapidly, while in slightly acidic to neutral environments (pH 6–7), the reduction of ketones and aldehydes is significantly slower. In either situation, however, the reduction of imines or iminium ions is known to be kinetically favored over the reduction of carbonyl groups [47,48]. Thus, the presence of the reduced products, TEMPOL **3** and the alcohol derivative of ciprofloxacin **4**, indicate an excessively acidic reaction environment conducive to the rapid reduction of ketones. The formation of the desired product **8** and the alcohol derivative **4** indicates successful iminium ion production and subsequent reduction. However, it can be concluded that the rate of iminium ion formation must be significantly slower than the rate of ketone reduction under these conditions. 

**Scheme 1:**
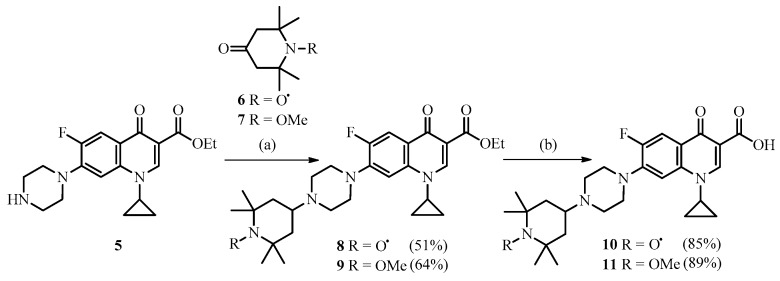
Synthetic route to ciprofloxacin-nitroxide hybrid **10** and its corresponding methoxyamine derivative **11**. *Reagent and conditions:* (**a**) (i) AcOH, MeOH 60 °C, 2 h. (ii) NaBH_3_CN, 50 °C, 24 h; (**b**) 2 M NaOH, MeOH, 50 °C, 5 h.

**Scheme 2:**
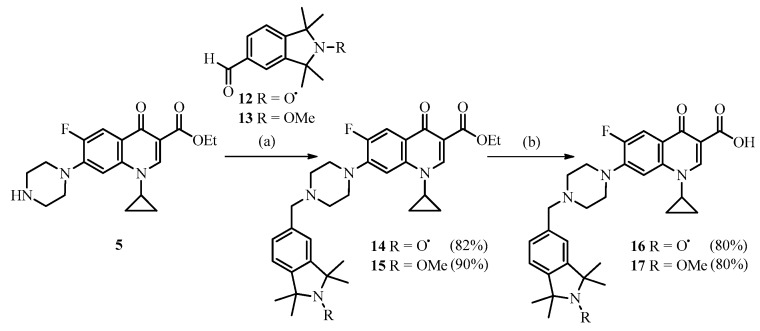
Synthetic route to ciprofloxacin-nitroxide hybrid **16** and its corresponding methoxyamine derivative **17**. *Reagent and conditions:* (**a**) (i) AcOH, MeOH 60 °C, 2 h. (ii) NaBH_3_CN, 50 °C, 24 h; (**b**) 2 M NaOH, MeOH, 50 °C, 5 h.

In order to circumvent the formation of the two undesired products, the reductive animation reaction was repeated using fewer equivalents of acetic acid (0.9 equivalents) and the iminium ion was generated by stirring at 60 °C for 2 h, prior to the addition of the reducing agent. Under these conditions, the desired ciprofloxacin-nitroxide hybrid **8** was isolated in reasonable yield (51%) after stirring at 50 °C overnight (Scheme 1). These optimized conditions were then employed to generate the conjugate compound **14** (Scheme 2) in an isolated yield of 82%. Final deprotection of ethyl esters (**8** and **14**) via base mediated hydrolysis produced the desired ciprofloxacin-nitroxide hybrids (**10** and **16**) in high yields (89% and 80% respectively, see supplementary materials for NMR and EPR spectra).

In addition to the generation of the novel ciprofloxacin-nitroxide hybrids (**10** and **16**), their methoxyamine derivatives (**11** and **17**) were also synthesised. These derivatives (**11** and **17**) were prepared as control compounds to enable a direct comparison of the biofilm dispersal effect of the nitroxide moiety. The methoxyamine functionality was introduced to the ketone and aldehyde functionalized nitroxides (**6** and **12**) utilising well-known Fenton chemistry [49]. The nitroxides (**6** and **12**) were treated with methyl radicals generated from hydrogen peroxide, iron(II) sulphate heptahydrate and DMSO [50] to furnish methoxyamines (**7** and **13**) in excellent yields (83% and 91% respectively). Reductive amination of methoxyamine derivatives (**7** and **13**) with the protected ciprofloxacin **5** utilising the methodology documented above gave the protected ciprofloxacin-methoxyamine conjugates (**9** and **15**) in good yields (64% and 90%, respectively). Subsequent ethyl ester deprotection of **9** and **15** generated the desired ciprofloxacin-methoxyamines (**11** and **17**) in high yields (85% and 80% respectively). 

### 2.2. Biological Testing

#### 2.2.1. Evaluation of Compounds **10**, **11**, **16** and **17** at 20 μM in *P. aeruginosa* Biofilms

**Figure 5:**
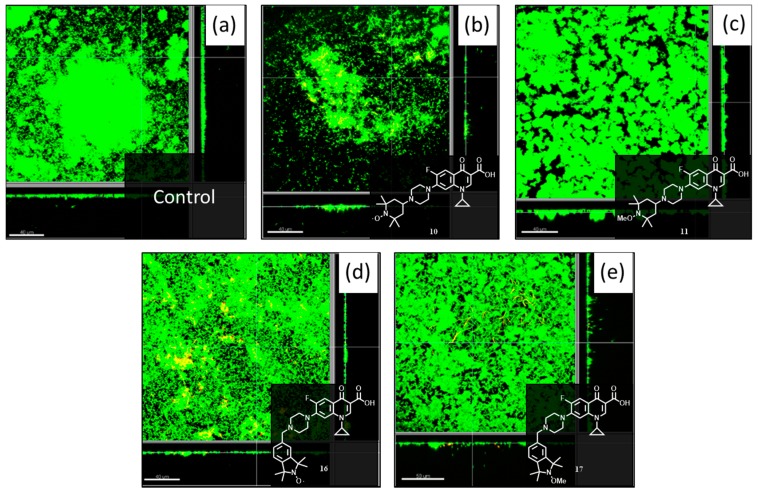
Confocal laser scanning microscopy images of 2 day old pre-formed *P. aeruginosa* PA14 biofilms grown in a flow cell at 37 °C, treated with 20 µM of (**b**) **10**; (**c**) **11**; (**d**) **16**; (**e**) **17** for 24 h and then visualized with SYTO-9 (stains live cells green) and propidium iodide (stains dead cells red, merged colors appear as yellow to red). Panel (**a**) shows an untreated *P. aeruginosa* PA14 biofilms after 3 days. The scale bars represent 40 µm in length for images (**a**–**c**,**e**), and 50 μm for image (**d**). Each panel also shows the xy, yz and xz dimensions.

Previously, our work has demonstrated that nitroxides can trigger dispersal events in pre-formed *P. aeruginosa* biofilms [27]. In addition to this, we have also shown that combined treatment with the nitroxide 4-carboxy-2,2,6,6-tetramethylpiperidin-1-yloxyl (CTEMPO) (at 20 µM) and the antibiotic ciprofloxacin **2** (at its MIC of 320 ng/mL (0.97 µM)) can result in almost complete eradication of mature *P. aeruginosa* and *E. coli* biofilms in a flow cell assay [31]. Here, we employed a similar approach involving pre-formed *P. aeruginosa* biofilms grown in flow cell chambers to evaluate the dispersal and eradication properties of the prepared ciprofloxacin-nitroxide compounds **10**, **11**, **16** and **17**. *P. aeruginosa* biofilms were formed in flow cell chambers for 48 h and then treated with 20 µM solutions of the hybrid compounds **10**, **11**, **16** and **17** (dissolved in DMSO and delivered into the BM2 minimal medium supplemented with 0.4% of glucose) for 24 h. This specific concentration was chosen as it was previously established to be the most effective concentration for nitroxide-mediated biofilm dispersal [27]. To aid in the visualization of the resulting biofilms the Live/Dead BacLight bacterial viability kit coupled with confocal microscopy was utilised and provided the images shown in Figure 5. From these images, the percentages of biofilm biomass eradicated using compounds **10**, **11**, **16** and **17** relative to the biomass of untreated 2-day-old biofilms were calculated and are displayed in Table 1. The values for the total live biofilm biomass eradication were calculated by adding the amount of dead cells remaining in the biofilm biomass to the initially eradicated biomass (i.e., compound **10** had initially 80% eradicated biomass but as 50% of the remaining 20% of biomass was dead, the total live biofilm biomass eradication was 90%). As a comparison, we observed little change in biofilm thickness by confocal laser scanning microscopy from the treatment of *P. aeruginosa* biofilms with ciprofloxacin alone (at 320 ng/mL) in our previous work [31].

The ciprofloxacin-nitroxide **10** and its methoxyamine derivative **11**, bearing the TEMPO moieties were examined first. In the presence of 20 μM of ciprofloxacin-nitroxide **10**, a significant reduction in the total biofilm biovolume (80%) was observed (Table 1, Figure 5b), however, 50% of the remaining biofilm biomass was composed of dead cells, which represents an overall reduction of 90% in live cell volume when compared to the untreated PA14 control (Table 1, Figure 5a). This is fairly consistent with our previous work, where we have observed that CTEMPO alone at 20 µM can disperse 2-day-old *P. aeruginosa* biofilms with a 60% reduction in total biofilm biovolume observed. The combination of CTEMPO at 20 µM and ciprofloxacin at 320 ng/mL (0.97 µM) caused a 99.3% reduction in total biofilm biovolume [31]. The corresponding ciprofloxacin-methoxyamine **11**, by comparison, was also able to reduce biofilm biomass by 30% at the same concentration (Table 1, Figure 5c), but only 5% of the remaining cells were dead in the final sample. This represents a total biofilm biomass reduction of only 34%, almost three times less than the nitroxide bearing conjugate **10**. Dispersal of mature *P. aeruginosa* biofilms by alkoxyamines has been previously observed by others for an ethoxyamine derivative in a crystal violet biofilm assay, however they also found the corresponding nitroxide to be more effective at inducing dispersal than the alkoxyamine derivative [28]. The flow cell assay results presented here clearly indicate that compound **10** possesses both dispersal and antibiotic activity, and this compound is the first example of a dual-action nitroxide-antibiotic hybrid molecule for the treatment of mature *P. aeruginosa* biofilms. 

Next the flow cell assay results from the ciprofloxacin-nitroxide compound **16** and its methoxyamine derivative **17** bearing the isoindoline units were analyzed. Both the nitroxide **16** and the methoxyamine derivative **17** (Figure 5d,e respectively), appear to trigger some dispersal and subsequent eradication of mature *P. aeruginosa* biofilms at 20 µM. The degree of eradication observed was slightly better for the nitroxide **16** (75% total biofilm biomass eradication) than the corresponding methoxyamine **17** (71% total biofilm biomass eradication), with nitroxide **16** able to kill a larger proportion of biofilm cells (32% of dead cells in remaining biomass) than the methoxyamine derivative **17** (23% of dead cells in remaining biomass). Overall, the best anti-biofilm agent at 20 μM was the hybrid compound **10**, which contains the TEMPO nitroxide unit. This result is consistent with our previous observation, which showed a higher degree of *P. aeruginosa* biofilm dispersal for the smaller TEMPO-based nitroxide over its isoindoline counterpart [27].

#### 2.2.2. Evaluation of Compounds **10**, **11**, **16** and **17** at 40 μM in *P. aeruginosa* Biofilms

**Figure 6:**
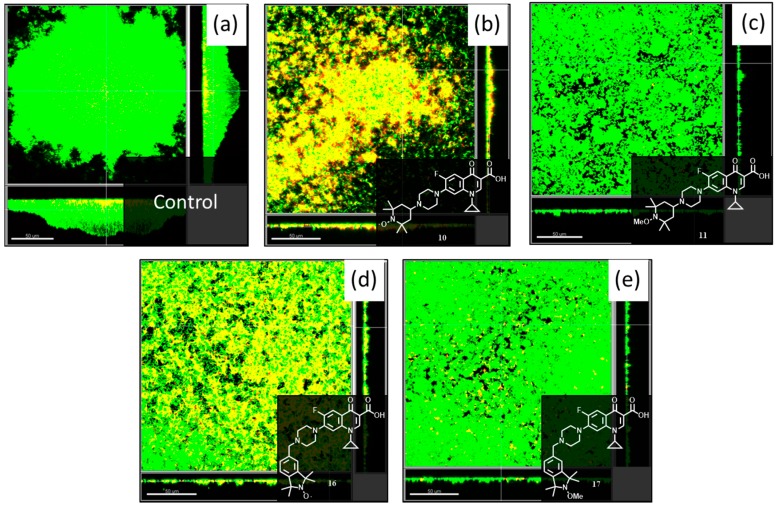
Confocal laser scanning microscopy images of 2 day old pre-formed *P. aeruginosa* PA14 biofilms grown in a flow cell at 37 °C, treated with 40 µM of (**b**) **10**; (**c**) **11**; (**d**) **16**; (**e**) **17** for 24 h and then visualized with SYTO-9 (stains live cells green) and propidium iodide (stains dead cells red, merged colors appear as yellow to red). Panel (**a**) shows an untreated *P. aeruginosa* PA14 biofilms after 3 days. The scale bars represent 50 µm in length for images (**a**–**d**). Each panel also shows the xy, yz and xz dimensions.

**Figure 7:**
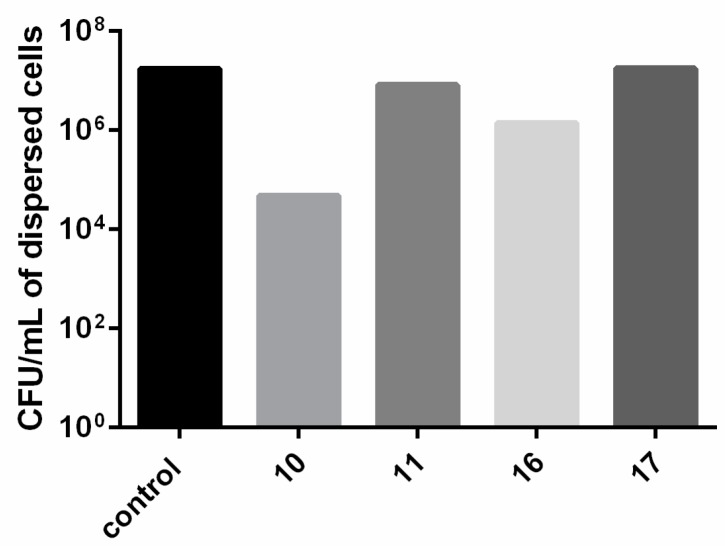
Flow cell effluent harvested from preformed *P. aeruginosa* PA14 biofilms treated with 40 µM of compounds **10**, **11**, **16**, and **17** after 24 h. Bacteria were plated for enumeration.

Although compounds **10**, **11**, **16** and **17** demonstrated biofilm eradication capabilities at a concentration of 20 μM, the anti-biofilm effects of the prepared compounds were also examined at the higher concentration of 40 μM. Treatment of 2 day old *P. aeruginosa* biofilms with compounds **10**, **11**, **16** and **17** at 40 μM for 24 h gave the images shown in Figure 6 (visualization was undertaken as previously described) from which values for eradication of the biofilm biomass could be calculated (Table 2). For this series of experiments, the viability of the dispersed cells was also quantified by collecting effluent from hybrid- and control- treated flow cells and is summarized in Figure 7.

Examination of the anti-biofilm activities of ciprofloxacin-nitroxide **10** and its methoxyamine conjugate **11** (bearing the TEMPO moieties), revealed that in the presence of 40 μM of hybrid **10**, a smaller reduction in total biofilm biovolume (41%) was observed, compared to ciprofloxacin-nitroxide **10** at 20 μM (80% biofilm reduction) (Figure 6b). However, for compound **10** at 40 μM, of the 59% remaining biomass, 91% was composed of dead cells, which corresponds to an overall reduction of 95% in live cell volume (a 5% improvement over compound **10** at 20 μM) when compared to the untreated PA14 control (Figure 6a). This result indicates that higher doses of hybrid compound **10** increased the potency of the compound against mature *P. aeruginosa* biofilms, but that removal of the majority of dead biofilm biomass in the flow cell did not occur. We speculate that this dead biomass contains various released cytoplasmic components (i.e., DNA) which contribute to its heightened adhesion in the flow cell. Nevertheless, fewer viable dispersed cells were collected from biofilms treated with compound **10** as compared to untreated biofilms or biofilms treated with all other compounds (Figure 7), reinforcing the potency of this hybrid. Interestingly, the methoxyamine derivative **11** performed only marginally better at a concentration of 40 μM (Figure 6c) than 20 μM (values of 40% and 34% respectively for total biofilm biomass eradication), which further demonstrates the fundamental importance of the nitroxide moiety to the anti-biofilm activity of hybrid compound **10**. Consistent with the minimal dead biomass, compound **11** did not affect the viability of dispersed bacteria (Figure 7).

Lastly, the effects of ciprofloxacin-nitroxide **16** and its methoxyamine conjugate **17** (containing the isoindoline moiety) in mature *P. aeruginosa* biofilms were analyzed at the higher concentration of 40 μM. Both the nitroxide bearing compound **16** and its methoxyamine derivative **17** reduced the biofilm biomass volume by the same amount (48%) (Figure 6d,e respectively), which was less than compounds **16** and **17** at 20 μM (63% and 62% respectively). However, the increased concentration (40 μM) improved the potency of compound **16** by increasing the total biofilm biomass eradication from 75% (20 μM) to 81% (40 μM). Comparatively, the methoxyamine conjugate **17** was only able to reduce the living cell population to 62%, which was less than the nitroxide containing compound **16**. Consistent with these findings, fewer viable dispersed cells were recovered from PA14 biofilms treated with compound **16** as compared to compound **17**. In general, biofilm eradication was not considerably enhanced at the higher concentration of hybrid molecules, however, the percentage of dead biofilm mass for each respective compound was greater. Future investigations will examine in more detail the observed anti-biofilm effects of ciprofloxacin-nitroxide conjugate molecules. 

## 3. Experimental Section

### 3.1. General Procedures

Reactions of an air-sensitive nature were carried out under an atmosphere of ultra-high purity argon. Where anhydrous THF, DMF, DCM or acetonitrile are documented, these solvents were obtained from a Puresolv Micro Multi Unit solvent purification system (Innovative Technologies Amesbury, MA, USA). Anhydrous toluene was dried by storage over sodium wire. Triethylamine and *i*-Pr_2_NEt were stored over potassium hydroxide. All other reagents were purchased from commercial suppliers and used without further purification. All ^1^H-NMR spectra were recorded at either 400 or 600 MHz on either a Varian Inova 400 spectrometer (Varian, Palo Alto, CA, USA), a Bruker Avance 400 spectrometer (Bruker, Billerica, MA, USA) or a Bruker Avance 600 spectrometer. All ^13^C-NMR spectra were recorded at either 100 or 150 on either a Varian Inova 400, a Bruker Avance 400 or a Bruker Avance 600 instrument. Samples were prepared in CDCl_3_, unless otherwise stated, using oven dried glassware. ^1^H-NMR spectra in CDCl_3_ were referenced to the solvent peak at 7.27 ppm. ^13^C-NMR spectra run in CDCl_3_ were referenced to the solvent peak at 77.2 ppm. Coupling constants are reported in Hz. High-resolution ESI mass spectra were obtained with an Agilent Q-TOF LC mass spectrometer (Agilent Technologies, Santa Clara, CA, USA) or a Thermo Fisher Scientific Orbitrap Elite mass spectrometer (Thermo Fisher Scientific, Waltham, MA, USA), which both utilised electrospray ionization in positive ion mode. Fourier transform infrared (FTIR) spectra were recorded on a Nicolet 870 Nexus Fourier Transform Infrared Spectrometer (Thermo Fisher Scientific) equipped with a DTGS TEC detector and an ATR objective. Melting points were measured with a Variable Temperature Apparatus by the capillary method and are uncorrected. Analytical HPLC was carried out on an Agilent Technologies HP 1100 Series HPLC system using an Agilent C18 column (4.6 × 250 mm, 5 μm) or an Agilent Zorbax RX-SIL column (4.6 × 250 mm, 5 μm) with a flow rate of 1 mL/min. The purity of all final compounds was determined to be 95% or higher using HPLC analysis. EPR spectra were obtained with the aid of a miniscope MS 400 Magnettech EPR spectrometer (Magnettech GmbH, Berlin, Germany). Column chromatography was performed using LC60A 40–63 Micron DAVISIL silica gel (Grace, Columbia, MD, USA). Thin-layer chromatography (TLC) was performed on Silica Gel 60 F254 plates (Merck, Billerica, MA, USA). TLC plates were visualised under a UV lamp (254 nm) and/or by visualization with phosphomolybdic acid (PMA).

### 3.2. Materials

Ethyl 1-cyclopropyl-6-fluoro-4-oxo-7-(piperazin-1-yl)-1,4-dihydroquinoline-3-carboxylate **5** [44], 1-methoxy-2,2,6,6-tetramethyl-4-piperidinone **7** [51] and 5-formyl-2-methoxy-1,1,3,3-tetramethylisoindoline **12** [49] were prepared according to known procedures.

### 3.3. Biofilm Dispersal Flow Cell Assays 

*P. aeruginosa* PA14 biofilms were pre-formed at 37 °C over 48 h in flow chambers using previously established techniques [27]. The biofilms were then exposed for 24 h to 20 or 40 µM solutions of ciprofloxacin-nitroxide hybrid compounds **10**, **11**, **16** and **17** resuspended in DMSO in the flow cell chambers with channel dimensions of 1 × 4 × 40 mm. Flow chambers were inoculated with 400 μL of an overnight *P. aeruginosa* PA14 culture diluted to an OD_600_ of ~0.05. Next, chambers were left without flow for 2 h, after which medium was pumped through the system at a constant rate of 2.4 mL/h. Staining and visualization of the resulting biofilms was performed using the Live/Dead BacLight bacterial viability kit (Thermo Fisher Scientific) and an Olympus Fluoview FV1000 confocal laser scanning microscope (Olympus, Center Valley, PA, USA). Three-dimensional reconstructions and residue biofilm biovolume calculations were done using Imaris software (Bitplane, Zurich, Switzerland). Untreated control samples were conducted in duplicate while compound-treated samples (at both 20 or 40 µM) were conducted once. Biovolume calculations were taken from three distinct fields of view for each flow cell chamber. Images that corresponded to the medium biovolume values were selected as representative images. To quantify dispersed bacteria, flow cell effluent was captured from flow cells treated with 40 µM of compounds **10**, **11**, **16** and **17** after 24 h. Bacteria were plated for enumeration. Enumeration of dispersal cells was conducted once. 

### 3.4. General Procedure for Reductive Amination, Compounds ***8***, ***9***, ***14*** and ***15***

Glacial acetic acid (0.9 equiv.) was added to a solution of ethyl ester protected ciprofloxacin **3** (1 equiv.) and the specific ketone/aldehyde bearing compound (1.5 equiv.) in anhydrous methanol under an atmosphere of argon. The resulting solution was stirred at 60 °C for 2 h and after cooling to room temperature, NaBH_3_CN (1 equiv.) was added followed by stirring at 50 °C for 1 day. The reaction mixture was diluted with deionized water (40 mL), the pH adjusted to ~7 using saturated aqueous sodium bicarbonate solution and the mixture extracted with dichloromethane (3 × 30 mL). The combined organic extracts were dried over anhydrous sodium sulfate and the solvent was removed in vacuo to afford the crude solid product. Purification was achieved using column chromatography (SiO_2_, eluent: 95% dichloromethane, 5% methanol).

*Ethyl 1-cyclopropyl-6-fluoro-7-(4-(2,2,6,6-tetramethyl-1-oxypiperidine-4-yl)piperazin-1-yl)-4-oxo-1,4-dihydroquinoline-3-carboxylate* **8**: Reagents: **5** (50 mg, 0.14 mmol, 1 equiv.), ketone **6** (35 mg, 0.21 mmol, 1.5 equiv.), glacial acetic acid (9 μL, 0.13 mmol, 0.9 equiv.), NaBH_3_CN (8 mg, 0.14 mmol, 1 equiv.) and HPLC grade methanol (10 mL). Data for **8**: Light beige powder (37 mg, 0.07 mmol, 51%); mp 212–222 °C decomposes. IR (ATR) ν_max_ (cm^−1^) = 1721 (s, C=O, ester), 1690 (s, C=O, ketone). ^1^H-NMR (600 MHz, CDCl_3_) δ = (*note compound is a free-radical, some signals appear broadened and other signals are missing) δ = 8.59 (s, 1H, NCH=C), 8.11 (d, *J* = 11.6 Hz, 1H, Ar-H), 7.36 (s, 1H, Ar-H), 4.46 (q, *J* = 6.6 Hz, 2H, OCH_2_CH_3_), 3.51 (br, s, 4H, 2 × NCH_2_ piperazyl ring), 3.36 (br, s, 1H, C=CHNCH cyclopropyl ring), 2.76 (br, s, 4H, 2 × NCH_2_ piperazyl ring), 1.48 (t, *J* = 6.8 Hz, 3H, OCH_2_CH_3_), 1.32 (br, s, 2H, NCHCH_2_ cyclopropyl ring), 1.22 (br, s, 2H, NCHCH_2_ cyclopropyl ring). ^13^C-NMR (150 MHz, CDCl_3_) δ = 172.1, 164.8, 153.2, 151.6, 147.1, 143.3, 137.0, 122.2, 112.4, 112.2, 109.5, 103.7, 60.3, 59.9, 53.5, 50.3, 48.3, 45.7, 33.6, 24.4, 22.2, 13.4, 7.2 HRMS (ESI): *m*/*z* calcd for C_28_H_38_FN_4_O_4_ + H^+^ [M + H]^+^: 514.2955. Found 514.2999. HPLC analysis: retention time = 4.719 min; peak area, 96.30%; eluent A, Methanol; eluent B, H_2_O; isocratic (80:20) over 20 min with a flow rate of 1 mL min^−1^ and detected at 254 nm; column temperature, rt. EPR: g = 1.9989, a_N_ = 1.5750 mT.

*Ethyl 1-cyclopropyl-6-fluoro-7-(4-(1-methoxy-2,2,6,6-tetramethylpiperidin-4-yl)piperazin-1-yl)-4-oxo-1,4-dihydroquinoline-3-carboxylate* **9**: Reagents: **5** (50 mg, 0.14 mmol, 1 equiv.), ketone **7** (35 mg, 0.21 mmol, 1.5 equiv.), glacial acetic acid (9 μL, 0.13 mmol, 0.9 equiv.), NaBH_3_CN (8 mg, 0.14 mmol, 1 equiv.) and HPLC grade methanol (10 mL). Data for **9**: White powder (45 mg, 0.09 mmol, 64%); mp 268–269 °C decomposed. IR (ATR) ν_max_ (cm^−1^) = 1722 (s, C=O, ester), 1617 (s, C=O, ketone). ^1^H-NMR (600 MHz, CDCl_3_) δ = 8.49 (s, 1H, NCH=C), 7.98 (d, *J* = 13.2 Hz, 1H, Ar-H), 7.25 (d, *J* = 7.0 Hz, 1H, Ar-H), 4.37 (q, *J* = 7.1 Hz, 2H, OCH_2_CH_3_), 3.61 (s, 3H, NOCH_3_), 3.61 (m, 1H, C=CHNCH cyclopropyl ring), 3.29 (br, s, 4H, 2 × NCH_2_ piperazyl ring), 2.81 (br, s, 4H, 2 × NCH_2_ piperazyl ring), 2.67 (br, s, NCHCH_2_ TEMPO ring), 1.68 (d, *J* = 11.6 Hz, 2H, NCHCH_2_ TEMPO ring), 1.54 (t, *J* = 12.0 Hz, 2H, NCHCH_2_ TEMPO ring), 1.39 (t, *J* = 6.7 Hz, 3H, OCH_2_CH_3_), 1.30 (q, *J* = 6.7 Hz, 2H, NCHCH_2_ cyclopropyl ring), 1.23 (s, 6H, 2 × CH_3_), 1.20 (s, 2H, NCHCH_2_ cyclopropyl ring), 1.14 (s, 6H, 2 × CH_3_). ^13^C-NMR (150 MHz, CDCl_3_) δ = 173.3, 166.0, 154.4, 152.7, 148.3, 144.7, 138.1, 123.2, 113.4, 113.3, 110.6, 105.0, 65.6, 63.3, 61.0, 60.0, 54.8, 50.4, 48.9, 48.4, 41.1, 34.6, 33.5, 33.2, 21.0, 20.8, 14.6, 8.3 HRMS (ESI): *m*/*z* calcd for C_29_H_41_FN_4_O + H^+^ [M + H]^+^: 529.3190. Found 529.3251. HPLC analysis: retention time = 14.443 min; peak area, 100%; eluent A, Methanol; eluent B, H_2_O; isocratic (80:20) over 20 min with a flow rate of 1 mL·min^−1^ and detected at 254 nm; column temperature, rt.

*Ethyl 1-cyclopropyl-6-fluoro-7-(4-(1,1,3,3-tetramethyl-2-oxyisoindolin-5-yl)methyl)piperazin-1-yl)-4-oxo-1,4-dihydroquinoline-3-carboxylate* **14**: Reagents: **5** (70 mg, 0.19 mmol, 1 equiv.), aldehyde **12** (63 mg, 0.29 mmol, 1.5 equiv.), glacial acetic acid (10 μL, 0.17 mmol, 0.9 equiv.), NaBH_3_CN (12 mg, 0.19 mmol, 1 equiv.) and HPLC grade methanol (10 mL). Data for **14**: Light beige powder (90 mg, 0.16 mmol, 82%); mp 210–211 °C. IR (ATR) ν_max_ (cm^−1^) =1724 (s, C=O, ester), 1613 (s, C=O, ketone). ^1^H-NMR (600 MHz, CDCl_3_) δ = (*note compound is a free-radical, some signals appear broadened and other signals are missing) δ = 8.57 (s, 1H, NCH=C), 8.09 (d, *J* = 12.7 Hz, 1H, Ar-H), 7.32 (s, 1H, Ar-H), 4.44 (q, 2H, OCH_2_CH_3_), 4.28 (br, s, 2H, NCH_2_Ar), 3.46 (m, 1H, C=CHNCH cyclopropyl ring) 3.38 (br, s, 4H, 2 × NCH_2_ piperazyl ring), 2.79 (br, s, 4H, 2 × NCH_2_ piperazyl ring), 1.46 (t, *J* = 6.8 Hz, 3H, OCH_2_CH_3_), 1.31 (br, s, 2H, NCHCH_2_ cyclopropyl ring), 1.19 (br, s, 2H, NCHCH_2_ cyclopropyl ring). ^13^C-NMR (150 MHz, CDCl_3_) δ = 172.3, 165.1, 153.8, 151.3, 147.3, 143.8, 137.2, 122.2, 112.6, 112.4, 109.6, 103.9, 60.1, 60.0, 49.2, 33.8, 13.6, 7.4. HRMS (ESI): *m*/*z* calcd for C_32_H_38_FN_4_O_4_ + H^+^ [M + H]^+^: 562.2950. Found 562.2950. HPLC analysis: retention time = 3.099 min; peak area, 99.70%; eluent A, Methanol; over 20 min with a flow rate of 1 mL·min^−1^ and detected at 254 nm; column temperature, rt. EPR: g = 1.9968, a_N_ = 1.5657 mT.

*Ethyl 1-cyclopropyl-6-fluoro-7-((4-(2-methoxy-1,1,3,3-tetramethylisoindoline-5-yl)methyl)piperazin-1-yl)-4-oxo-1,2,3,4-tetrahydroquinoline-3-carboxylate* **15**: Reagents: **5** (70 mg, 0.19 mmol, 1 equiv.), aldehyde **13** (68 mg, 0.29 mmol, 1.5 equiv.), glacial acetic acid (10 μL, 0.17 mmol, 0.9 equiv.), NaBH_3_CN (12 mg, 0.19 mmol, 1 equiv.) and HPLC grade methanol (10 mL). Data for **15**: White powder (99 mg, 0.17 mmol, 90%); mp 191–192 °C. IR (ATR) ν_max_ (cm^−1^) =1721 (s, C=O, ester), 1615 (s, C=O, ketone). ^1^H-NMR (600 MHz, CDCl_3_) δ = 8.51 (s, 1H, NCH=C), 8.02 (d, *J* = 13.3 Hz, 1H, Ar-H), 7.23 (m, 2H, Ar-H), 7.05 (m, 2H, Ar-H), 4.38 (q, *J* = 7.1 Hz, 2H, OCH_2_CH_3_), 3.79 (s, 3H, NOCH_3_), 3.59 (s, 2H, NCH_2_Ar), 3.41 (m, 1H, C=CHNCH cyclopropyl ring), 3.29 (s, 4H, 2 × NCH_2_ piperazyl ring), 2.67 (s, 4H, 2 × NCH_2_ piperazyl ring), 1.44 (br, s, 12H, 4 × CH_3_), 1.41 (t, *J* = 7.1 Hz, OCH_2_CH_3_), 1.29 (m, 2H, NCHCH_2_ cyclopropyl ring), 1.13 (m, 2H, NCHCH_2_ cyclopropyl ring). ^13^C-NMR (100 MHz, CDCl_3_) δ = 173.4, 166.1, 148.3, 145.5, 144.9, 144.5, 138.2, 136.9, 128.4, 123.2, 123.1, 122.4, 121.5, 113.6, 113.3, 110.6, 104.8, 67.2, 67.1, 65.7, 63.3, 61.1, 53.0, 50.2, 34.6, 14.6, 8.3. HRMS (ESI): *m*/*z* calcd for C_33_H_41_FN_4_O_4_ + H^+^ [M + H]^+^:577.3185. Found 577.3186. HPLC analysis: retention time = 4.092 min; peak area, 100%; eluent A, Methanol; over 20 min with a flow rate of 1 mL·min^−1^ and detected at 254 nm; column temperature, rt.

### 3.5. General Procedure for Ester Hydrolysis, Compounds ***10***, ***11***, ***16*** and ***17***

Aqueous sodium hydroxide (2 M, 7 equiv.) was added to a solution of the specific ethyl ester (1 equiv.) in HPLC grade methanol and the resulting solution was stirred at 50 °C for 5 h. The reaction mixture was cooled to room temperature and diluted with deionized water (50 mL). The pH was adjusted to ~6 using aqueous hydrochloric acid solution (2 M) and the mixture extracted with dichloromethane (3 × 20 mL). The combined organic extracts were dried over anhydrous sodium sulfate and the solvent was removed in vacuo to afford the pure solid product.

*1-Cyclopropyl-6-fluoro-7-(4-(2,2,6,6-tetramethyl-1-oxypiperidin-4-yl)piperazin-1-yl)-4-oxo-1,4-dihydroquinoline-3-carboxylic acid* **10**: Reagents: **8** (22 mg, 0.04 mmol, 1 equiv.), 2 M aqueous NaOH (0.1 mL, 0.2 mmol, 7 equiv.) and HPLC grade methanol (2.5 mL). Data for **10**: Light yellow powder (17 mg, 0.035 mmol, 89%); mp. 207–208 °C, decomposes. IR (ATR) ν_max_ (cm^−1^) = 3100–2500 (w, br, O-H, COOH). ^1^H-NMR (600 MHz, CDCl_3_) δ = (*note compound is a free-radical, some signals appear broadened and other signals are missing) 14.93 (s, 1H, COOH), 8.83 (s, 1H, NCH=C), 8.07 (d, *J* = 10.7 Hz, 1H, Ar-H), 7.47 (s, 1H, Ar-H), 3.62 (br, s, 4H, 2 × NCH_2_ piperazyl ring), 3.45 (m, 1H, C=CHNCH cyclopropyl ring), 2.80 (br, s, 4H, 2 × NCH_2_ piperazyl ring), 1.50 (br, s, 2H, NCHCH_2_ cyclopropyl ring), 1.31 (br, s, 2H, NCHCH_2_ cyclopropyl ring). ^13^C-NMR (150 MHz, CDCl_3_) δ = 175.5, 165.5, 153.0, 151.3, 145.9, 144.2, 137.6, 118.3, 110.9, 110.8, 106.6, 103.3, 47.7, 45.1, 34.1, 28.2, 6.9. HRMS (ESI): *m*/*z* calcd for C_26_H_34_FN_4_O_4_+ H^+^ [M + H]^+^: 486.2642. Found 486.2659. HPLC analysis: retention time = 4.569 min; peak area, 100%; eluent A, DCM; eluent B, THF; isocratic (70:30) over 20 min with a flow rate of 1 mL min^−1^ and detected at 254 nm; column temperature, rt. EPR: g = 1.9989, a_N_ = 1.5750 mT.

*1-Cyclopropyl-6-fluoro-7-(4-(1-methoxy-2,2,6,6-tetramethylpiperidin-4-yl)piperazin-1-yl)-4-oxo-1,4-dihydroquinoline-3-carboxylic acid* **11**: Reagents: **9** (20 mg, 0.04 mmol, 1 equiv.), 2 M aqueous NaOH (0.1 mL, 0.2 mmol, 7 equiv.) and HPLC grade methanol (2.5 mL). Data for **11**: White powder (17 mg, 0.03 mmol, 85%); mp 230–232 °C, decomposes. IR (ATR) ν_max_ (cm^−1^) = 3100-2500 (w, br, O-H, COOH). ^1^H-NMR (600 MHz, CDCl_3_) δ = 15.02 (s, 1H, COOH), 8.74 (s, 1H, NCH=C), 7.98 (d, *J* = 13.0 Hz, 1H, Ar-H), 7.35 (d, *J* = 7.0 Hz, 1H, Ar-H), 3.61 (s, 3H, NOCH_3_), 3.55 (m, 1H, C=CHNCH cyclopropyl ring), 3.34 (m, 4H, 2 × NCH_2_ piperazyl ring), 2.79 (m, 4H, 2 × NCH_2_ piperazyl ring), 2.75 (t, *J* = 3.3 Hz, NCHCH_2_ TEMPO ring), 1.66 (d, *J* = 12.6 Hz, 2H, NCHCH_2_ TEMPO ring), 1.52 (t, *J* = 12.2 Hz, 2H, NCHCH_2_ TEMPO ring), 1.39 (q, *J* = 6.5 Hz, 2H, NCHCH_2_ cyclopropyl ring), 1.23 (s, 6H, 2 × CH_3_), 1.20 (m, 2H, NCHCH_2_ cyclopropyl ring), 1.15 (s, 6H, 2 × CH_3_). ^13^C-NMR (150 MHz, CDCl_3_) δ = 175.5, 165.5, 153.0, 151.3, 145.9, 144.2, 137.6, 118.3, 111.1, 110.8, 106.6, 103.3, 47.7, 45.1, 34.1, 28.2, 25.4, 6.9. HRMS (ESI): *m*/*z* calcd for C_27_H_37_FN_4_O_4_ + H^+^ [M + H]^+^: 501.2877. Found 501.2857. HPLC analysis: retention time = 4.223 min; peak area, 100%; eluent A, DCM; eluent B, THF; isocratic (70:30) over 20 min with a flow rate of 1 mL·min^−1^ and detected at 254 nm; column temperature, rt. 

*1-Cyclopropyl-6-fluoro-7-((4-(1,1,3,3-tetramethyl-2-oxyisoindolin-5-yl)methyl)piperazin-1-yl)-4-oxo-1,2,3,4-tetrahydroquinoline-3-carboxylic acid* **16**: Reagents: **14** (50 mg, 0.089 mmol, 1 equiv.), 2 M aqueous NaOH (0.3 mL, 0.62 mmol, 7 equiv.) and HPLC grade methanol (2.5 mL). Data for **16**: Yellow powder (38 mg, 0.071 mmol, 80%); mp 230 °C, decomposes. IR (ATR) ν_max_ (cm^−1^) = 3100–2500 (w, br, O-H, COOH). ^1^H-NMR (600 MHz, CD_2_Cl_2_) (*note compound is a free-radical, some signals appear broadened and other signals are missing) δ = 13.73 (s, 1H, COOH), 8.76 (s, 1H, NCH=C), 8.03 (br, s, 1H, Ar-H), 7.42 (br, s, 1H, Ar-H), 4.44 (br, s, 2H, NCH_2_Ar), 3.92 (br, s, 2H, NCH_2_ piperazyl ring), 3.55 (br, s, 2H, NCH_2_ piperazyl ring), 3.38 (br, s, 2H, NCH_2_ piperazyl ring), 3.14 (s, 1H, C=CHNCH cyclopropyl ring), 2.72 (br, s, 2H, NCH_2_ piperazyl ring), 1.38 (s, 2H, NCHCH_2_ cyclopropyl ring), 1.19 (s, 2H, NCHCH_2_ cyclopropyl ring). ^13^C-NMR (150 MHz, CD_2_Cl_2_) δ = 177.2, 35.4, 8.3. HRMS (ESI): *m*/*z* calcd for C_30_H_34_FN_4_O_4_ + H^+^ [M + H]^+^: 534.2637. Found 534.2639. HPLC analysis: retention time = 3.801 min; peak area, 100%; eluent A, CH_3_CN; eluent B, H_2_O/TFA (99:1); isocratic (95:5) over 20 min with a flow rate of 1 mL·min^−1^ and detected at 254 nm; column temperature, rt. EPR: g = 1.9997, a_N_ = 1.4924 mT.

*1-Cyclopropyl-6-fluoro-7-((4-(2-methoxy-1,1,3,3-tetramethylisoindolin-5-yl)methyl)piperazin-1-yl)-4-oxo-1,2,3,4-tetrahydroquinoline-3-carboxylic acid* **17**: Reagents: **15** (50 mg, 0.087 mmol, 1 equiv.), 2 M aqueous NaOH (0.30 mL, 0.61 mmol, 7 equiv.) and HPLC grade methanol (2.5 mL). Data for **17**: White powder (40 mg, 0.072 mmol, 83%); mp 299 °C, decomposes. IR (ATR) ν_max_ (cm^−1^) = 3100–2500 (w, br, O-H, COOH). ^1^H-NMR (600 MHz, CD_2_Cl_2_) δ = 13.45 (s, 1H, COOH), 8.78 (s, 1H, NCH=C), 8.04 (d, *J* = 12.8 Hz, 1H, Ar-H), 7.56 (d, *J* = 7.1 Hz, 1H, Ar-H), 7.48 (m, 2H, Ar-H), 7.22 (d, *J* = 12.8 Hz, 1H, Ar-H), 4.22 (d, *J* = 4.7 Hz, 2H, NCH_2_Ar), 3.89 (m, 2H, NCH_2_ piperazyl ring), 3.79 (s, 3H, NOCH_3_), 3.73 (m, 2H, NCH_2_ piperazyl ring), 3.56 (m, 1H, C=CHNCH cyclopropyl ring), 3.53 (s, 2H, 2 × NCH_2_ piperazyl ring), 3.08 (m, 2H, 2 × NCH_2_), 1.55 (br, s, 12H, 4 × CH_3_), 1.39 (m, 2H, 2 × NCHCH_2_ cyclopropyl ring), 1.18 (m, 2H, NCHCH_2_ cyclopropyl ring). ^13^C-NMR (150 MHz, CD_2_Cl_2_) δ = 166.9, 148.5, 131.4, 125.6, 123.0, 113.1, 107.1, 65.9, 61.7, 51.8, 47.1, 36.1, 8.8. HRMS (ESI): *m*/*z* calcd for C_31_H_37_FN_4_O_4_ + H^+^ [M + H]^+^: 549.2872. Found 549.2870. HPLC analysis: retention time = 4.304 min; peak area, 100%; eluent A, CH_3_CN; eluent B, H_2_O/TFA (99:1); isocratic (95:5) over 20 min with a flow rate of 1 mL·min^−1^ and detected at 254 nm; column temperature, rt.

## 4. Conclusions 

Two ethyl ester protected ciprofloxacin-nitroxide hybrids **8** and **14** together with their methoxyamine analogues **9** and **15** were prepared using reductive amination in moderate to high yield (51%–90%) from the corresponding ketone or aldehyde bearing nitroxides **6** and **12** or methoxyamines **7** and **13** and the ethyl ester protected ciprofloxacin **5**. Base mediated deprotection of **8**, **9**, **14** and **15** gave the desired active ciprofloxacin-nitroxides **10** and **16** and their control methoxyamines **11** and **17** in high yield (80%–90%). Biological evaluation of hybrid compounds **10**, **11**, **16** and **17** for anti-biofilm activity against mature *P. aeruginosa* biofilms was performed in a flow cell assay. Both of the ciprofloxacin-nitroxide hybrids were found to have the desired dual-action effect against established biofilms, with the most significant of these being compound **10**, which demonstrated both dispersal of *P. aeruginosa* biofilms and eradication (up to 95% at 40 µM) of the newly dispersed bacteria. In comparison, far less eradication (40% at 40 μM) of the established *P. aeruginosa* biofilm was observed following treatment with the corresponding methoxyamine **11**, suggesting that the nitroxide moiety is key to the effectiveness of analogue **10**. The results presented here demonstrate that the combination of an antibiotic and a nitroxide within a single molecule is potentially an effective approach to facilitate the efficient eradication of mature biofilms and thereby overcome the resistance of biofilms to antimicrobials. More detailed investigations into the observed anti-biofilm activities of the ciprofloxacin-nitroxide conjugates are currently in progress and will be reported in due course.

## Figures and Tables

**Table 1:** *P. aeruginosa* PA14 biofilm biomass eradication values for 2-day-old biofilms treated with compounds **10**, **11**, **16** and **17** at 20 µM relative to the biomass of untreated 2-day-old biofilms.

Compound	Remaining Biomass (%)	Dead Cells in Remaining Biomass (%)	Total Live Biofilm Biomass Eradication (%)
Control PA14	--	4	--
**10**	20	50	90
**11**	70	5	34
**16**	37	32	75
**17**	38	23	71

**Table 2:** *P. aeruginosa* PA14 biofilm biomass eradication values for 2-day-old biofilms treated with compounds **10, 11, 16** and **17** at 40 µM relative to the biomass of untreated 2-day-old biofilms.

Compound	Remaining Biomass (%)	Dead Cells in Remaining Biomass (%)	Total Live Biofilm Biomass Eradication (%)
Control PA14	--	4	--
**10**	59	91	95
**11**	65	7	40
**16**	52	64	81
**17**	52	27	62

## Supplementary Materials

Supplementary materials can be accessed at: http://www.mdpi.com/1420-3049/21/7/841/s1.

## Abbreviations

The following abbreviations are used in this manuscript:

Cyclic di-GMP: bis-(3′-5′)-cyclic dimeric guanosine monophosphate
DCM: dichloromethane
DMSO: dimethyl sulfoxide
DNA: deoxyribonucleic acid
EPR: electron paramagnetic resonance
NMR: nuclear magnetic resonance
NO: nitric oxide
THF: tetrahydrofuran
TLC: solid thin layer chromatography
