# Supplementary material for: Synthesis and Evaluation of Ciprofloxacin-Nitroxide Conjugates as Anti-Biofilm Agents
Source: Molecules. 2016 Jun 27;21(7):841. doi: 10.3390/molecules21070841 (PMC6273952; doi:10.3390/molecules21070841)
Supplement: Supplementary file 1 [file molecules-21-00841-s001.pdf]

Supplementary Materials: Synthesis and Evaluation of Ciprofloxacin-Nitroxide Conjugates as Anti-Biofilm Agents

Anthony D. Verderosa, Sarah C. Mansour, César de la Fuente-Núñez, Robert E. W. Hancock and Kathryn E. Fairfull-Smith

Table of Contents

Figure S1. ^{1}H-NMR spectrum of **8** S2
Figure S2. ^{13}C-NMR spectrum of **8** S2
Figure S3. HPLC chromatogram of **8** S3
Figure S4. EPR spectrum of **8** S3
Figure S5. ^{1}H-NMR spectrum of **9** S4
Figure S6. ^{13}C-NMR spectrum of **9** S4
Figure S7. HPLC chromatogram of **9** S5
Figure S8. ^{1}H-NMR spectrum of **10** S5
Figure S9. ^{13}C-NMR spectrum of **10** S6
Figure S10. HPLC chromatogram of **10** S6
Figure S11. EPR spectrum of **10** S7
Figure S12. ^{1}H-NMR spectrum of **11** S7
Figure S13. ^{13}C-NMR spectrum of **11** S8
Figure S14. HPLC chromatogram of **11** S8–S9
Figure S15. ^{1}H-NMR spectrum of **14** S9
Figure S16. ^{13}C-NMR spectrum of **14** S10
Figure S17. HPLC chromatogram of **14** S10
Figure S18. EPR spectrum of **14** S11
Figure S19. ^{1}H-NMR spectrum of **15** S11
Figure S20. ^{13}C-NMR spectrum of **15** S12
Figure S21. HPLC chromatogram of **15** S12
Figure S22. ^{1}H-NMR spectrum of **16** S13
Figure S23. ^{13}C-NMR spectrum of **16** S13
Figure S24. HPLC chromatogram of **16** S14
Figure S25. EPR spectrum of **16** S14
Figure S26. ^{1}H-NMR spectrum of **17** S15
Figure S27. ^{13}C-NMR spectrum of **17** S15
Figure S28. HPLC chromatogram of **17** S16

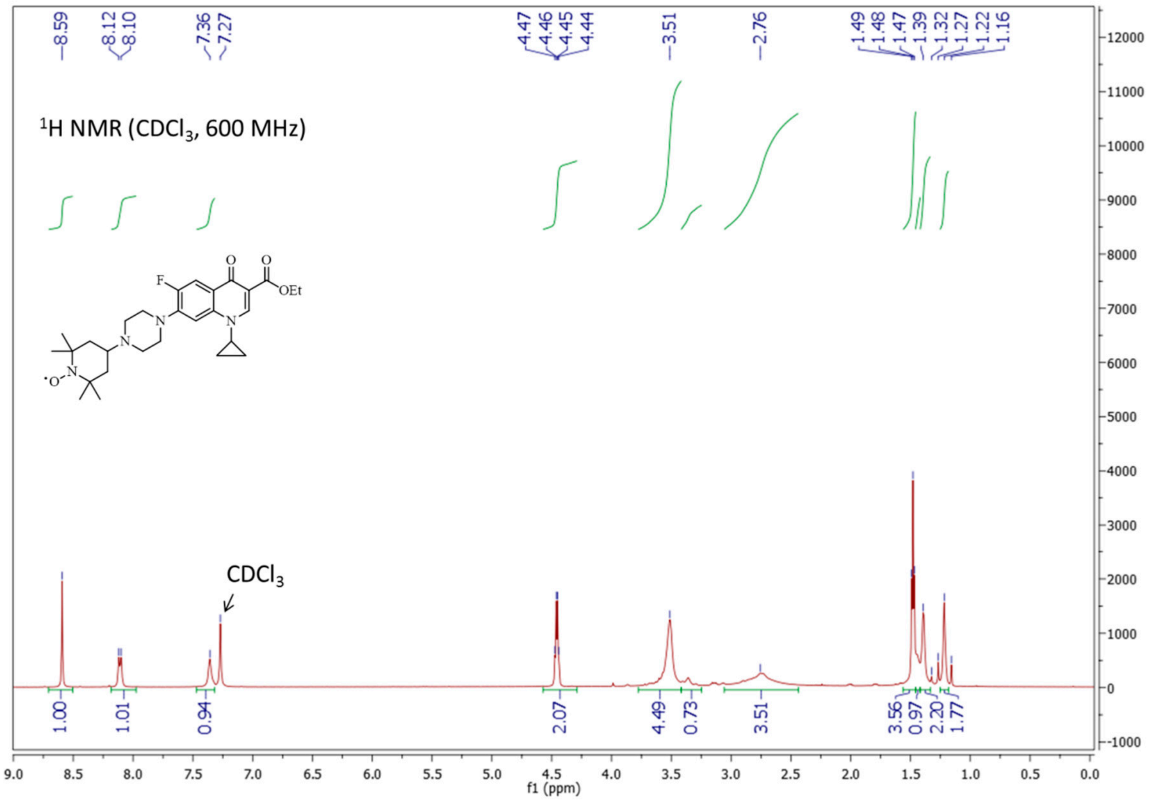

Figure S1. ^{1}H-NMR spectrum of **8**.

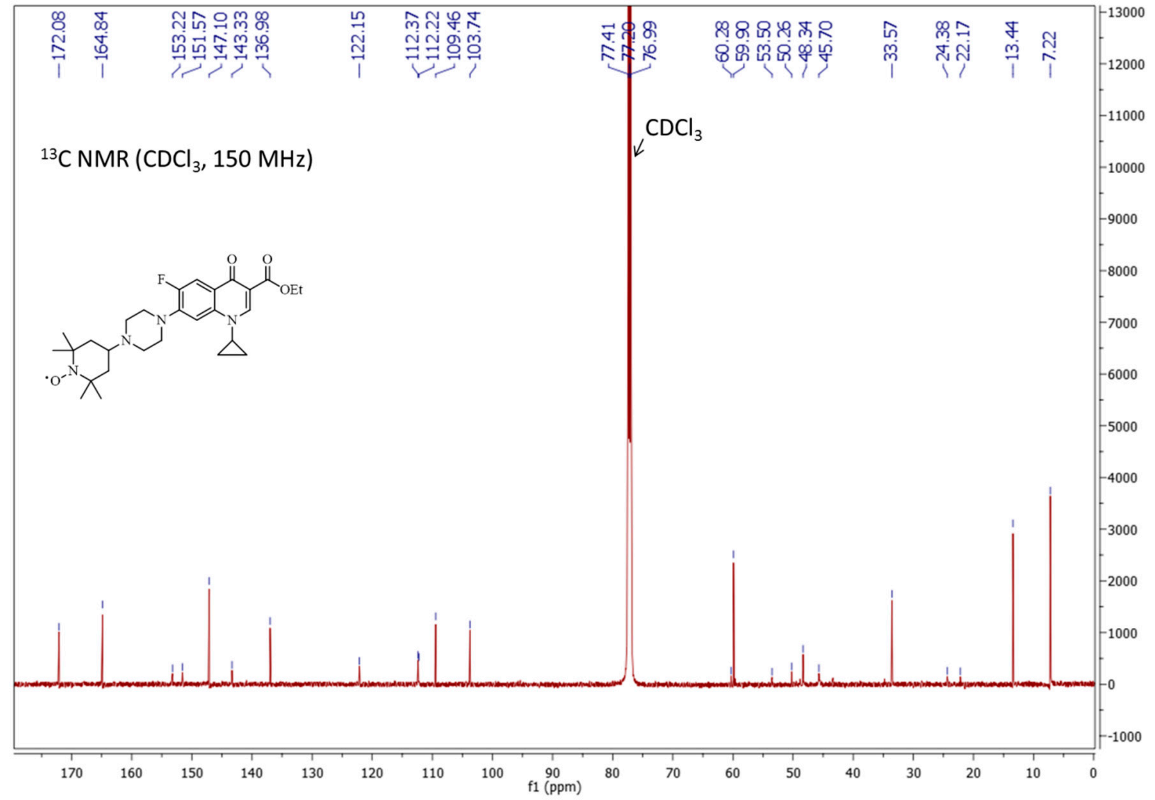

Figure S2. ^{13}C-NMR spectrum of **8**.

Column Type: Agilent C18 column (4.6 × 250 mm, 5 µm).
Flow Rate: 1 mL/min.
Solvent Composition: (MeOH:H_2O, 80:20).

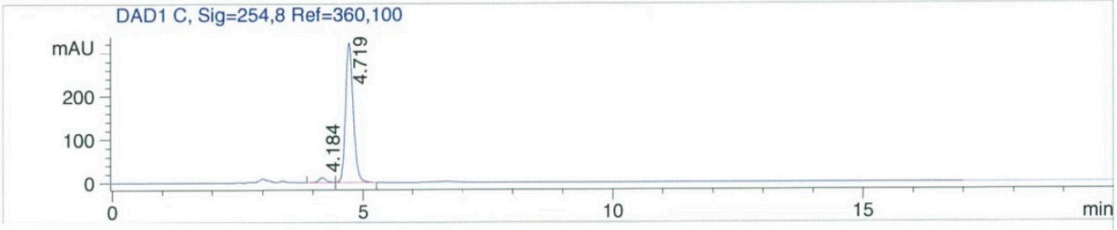

Peak No.	Area (%)	Retention Time (minutes)
1	3.700	4.184
2	96.300	4.719

Figure S3. HPLC chromatogram of **8**.

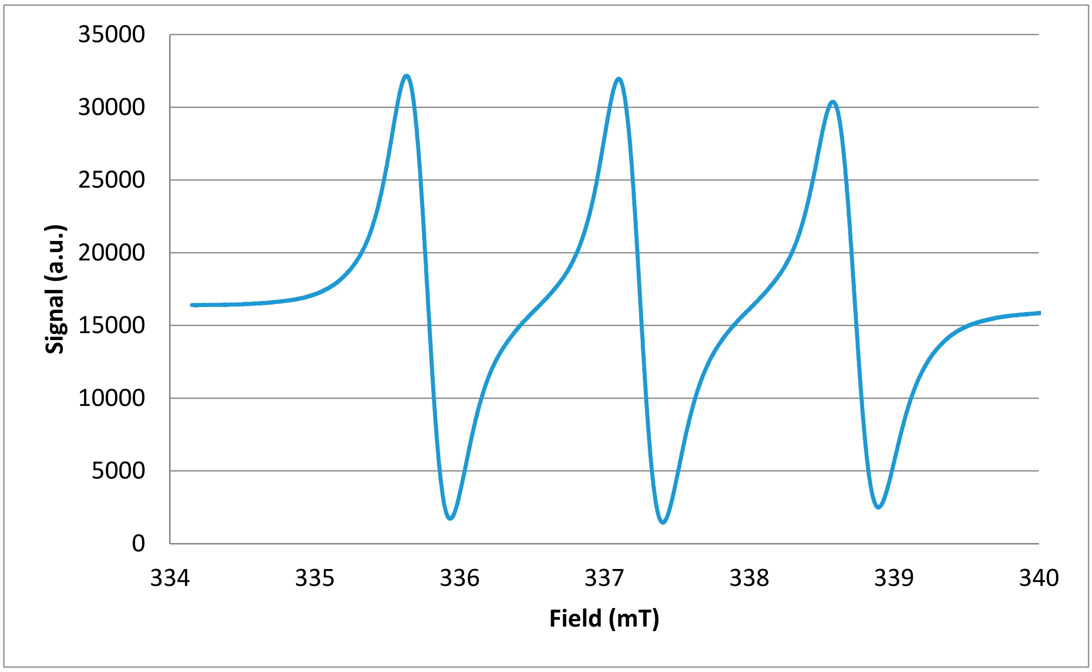

Figure S4. EPR spectrum of **8**.

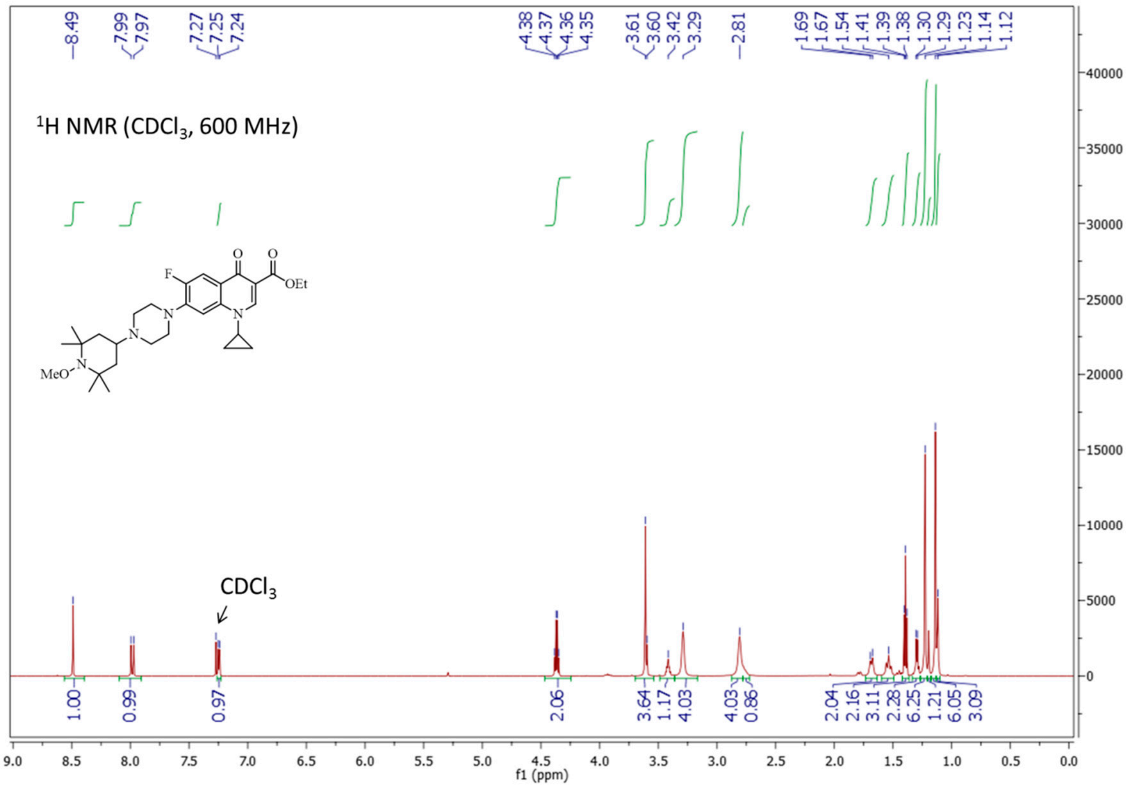

Figure S5. ^{1}H-NMR spectrum of **9**.

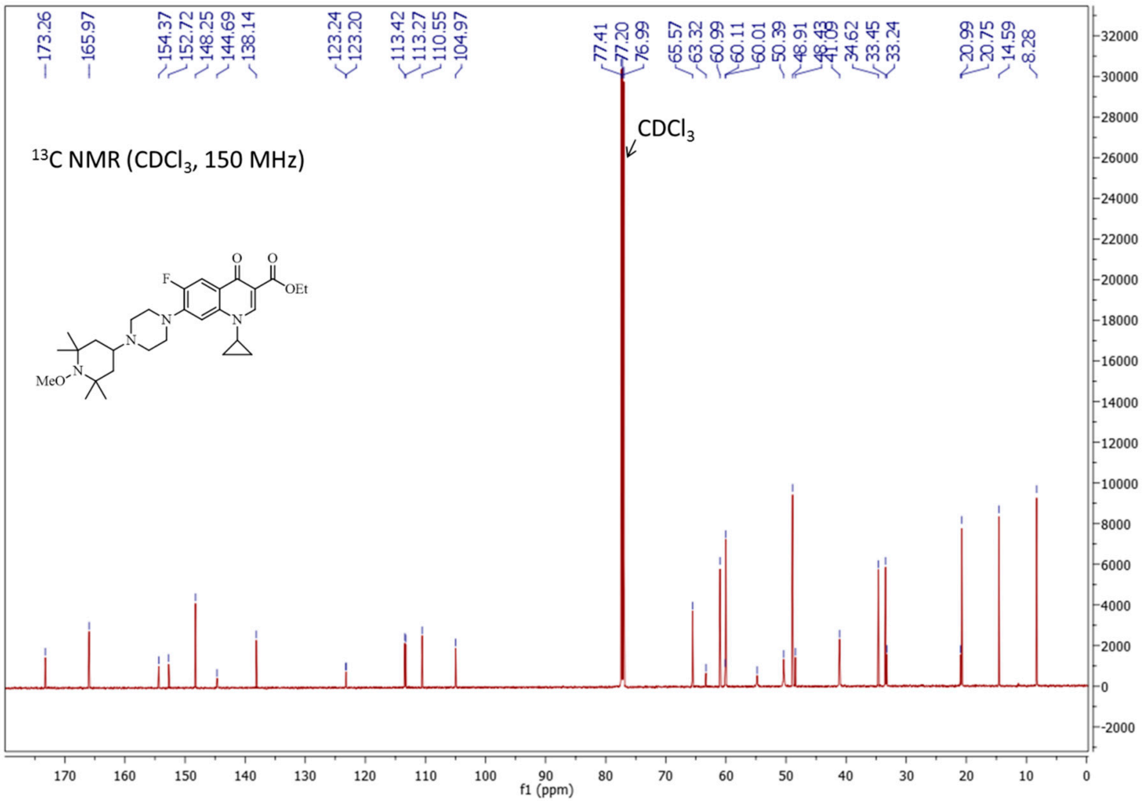

Figure S6. ^{13}C-NMR spectrum of **9**.

Column Type: Agilent C18 column (4.6 × 250 mm, 5 μm).
Flow Rate: 1 mL/min.
Solvent Composition: (MeOH:H_2O, 80:20).

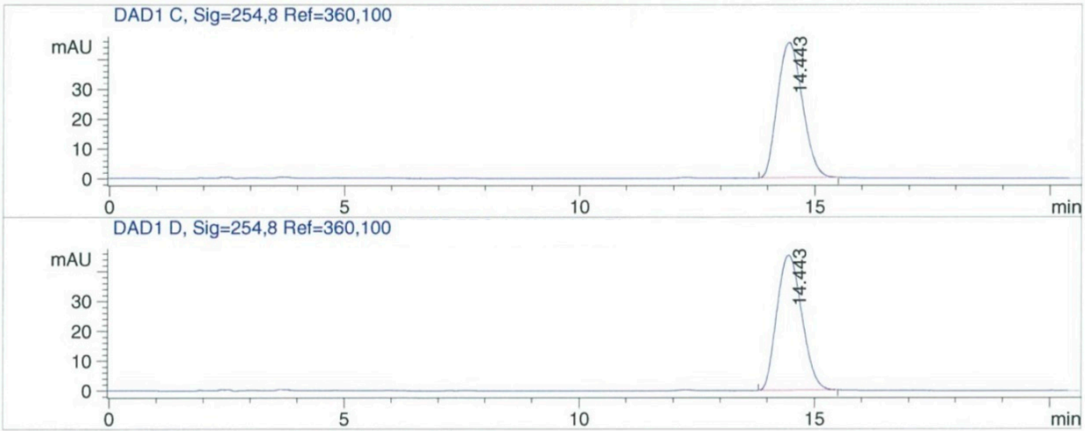

Peak No.	Area (%)	Retention Time (minutes)
1	100.000	14.443

Figure S7. HPLC chromatogram of **9**.

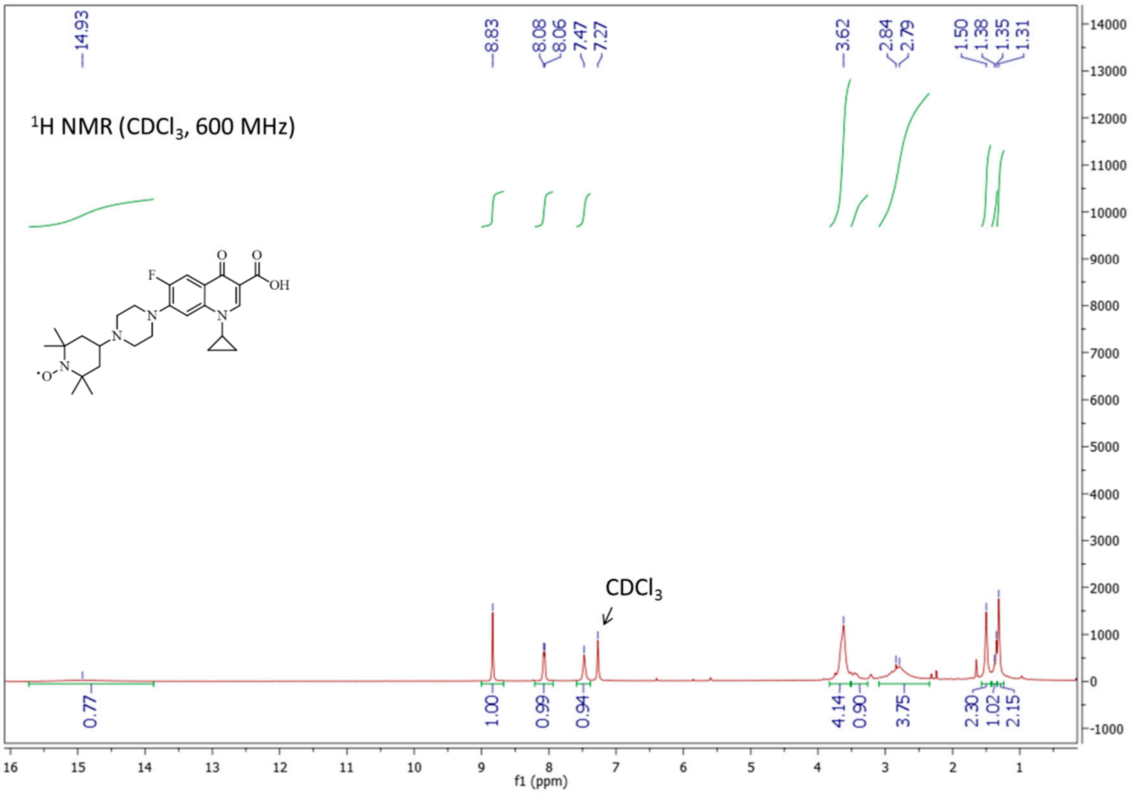

Figure S8. ^{1}H-NMR spectrum of **10**.

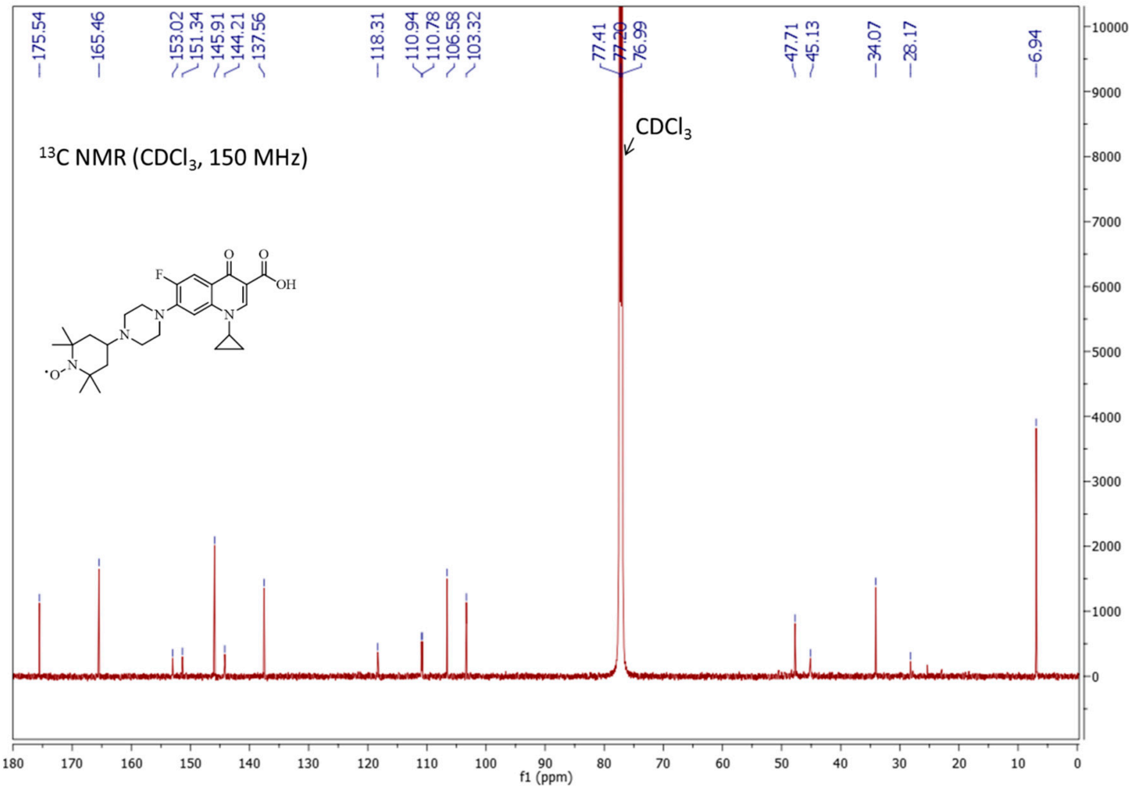

Figure S9. ^{13}C-NMR spectrum of **10**.

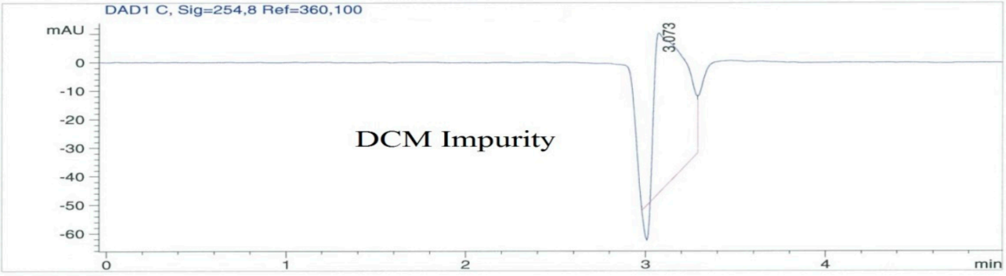

Peak No.	Retention Time (minutes)	Area	Main Pe
1	3.073	617.865	100.000

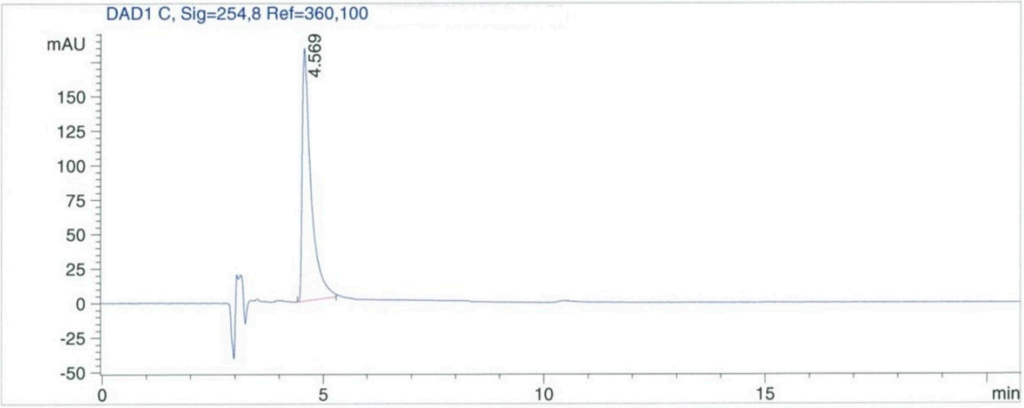

Peak No.	Retention Time (minutes)	Area	Main Pe
1	4.569	2518.024	100.000

Figure S10. HPLC chromatogram of **10**.

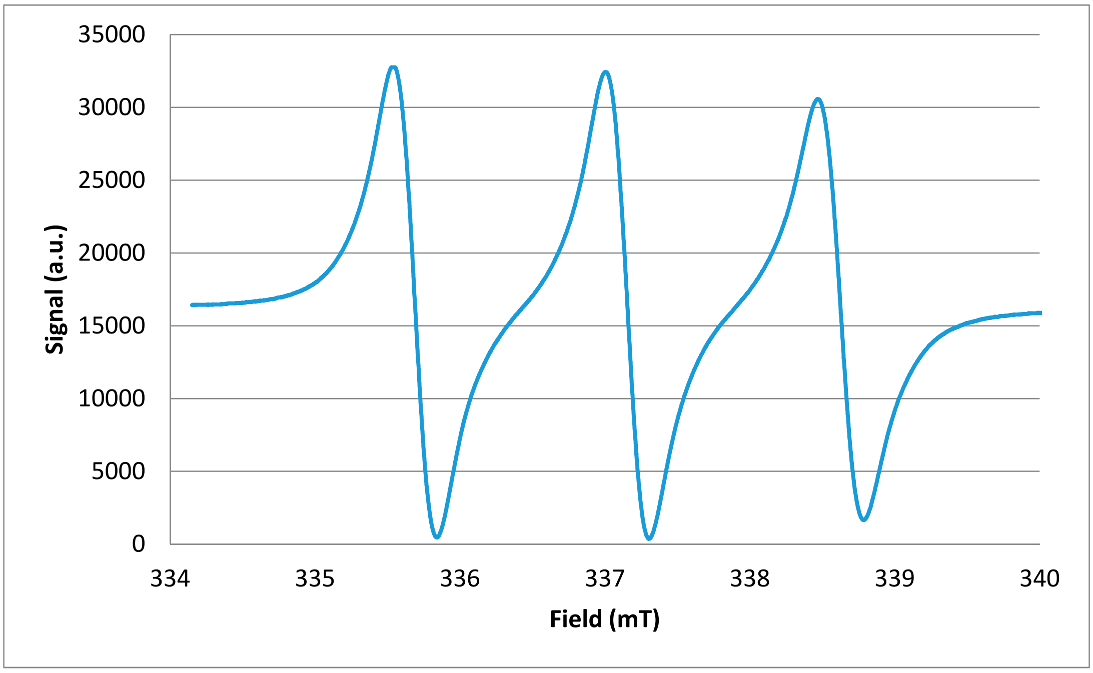

Figure S11. EPR spectrum of **10.**

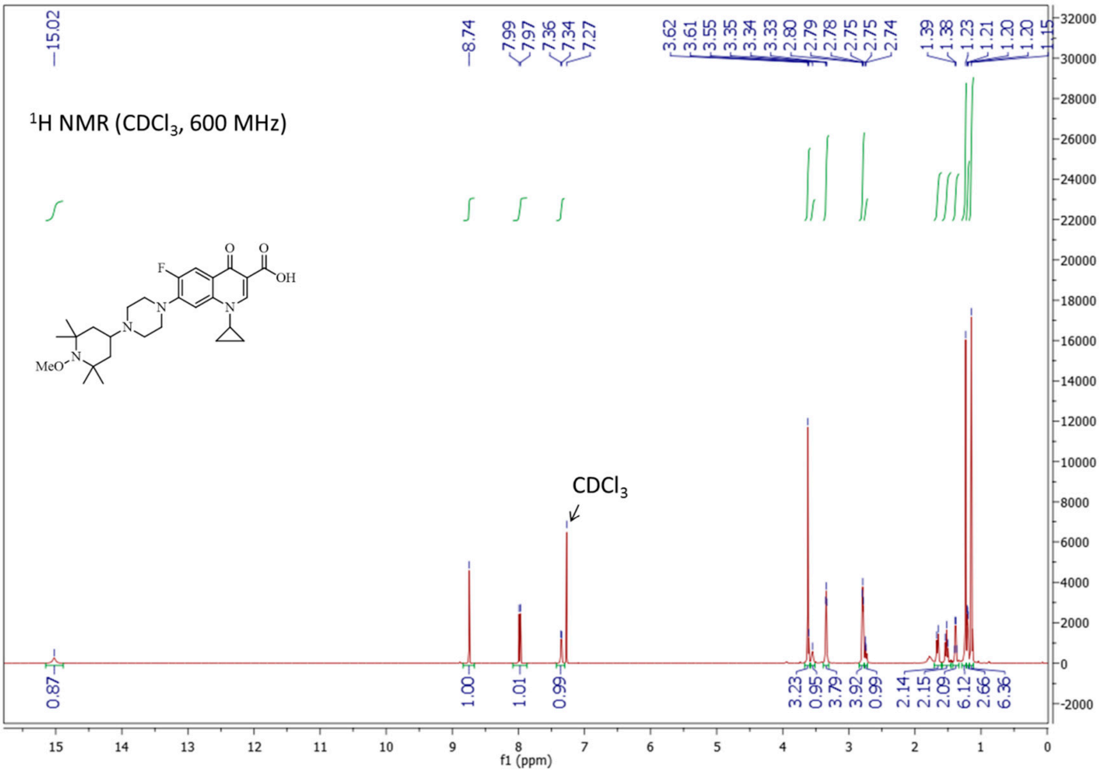

Figure S12. ^{1}H-NMR spectrum of **11**.

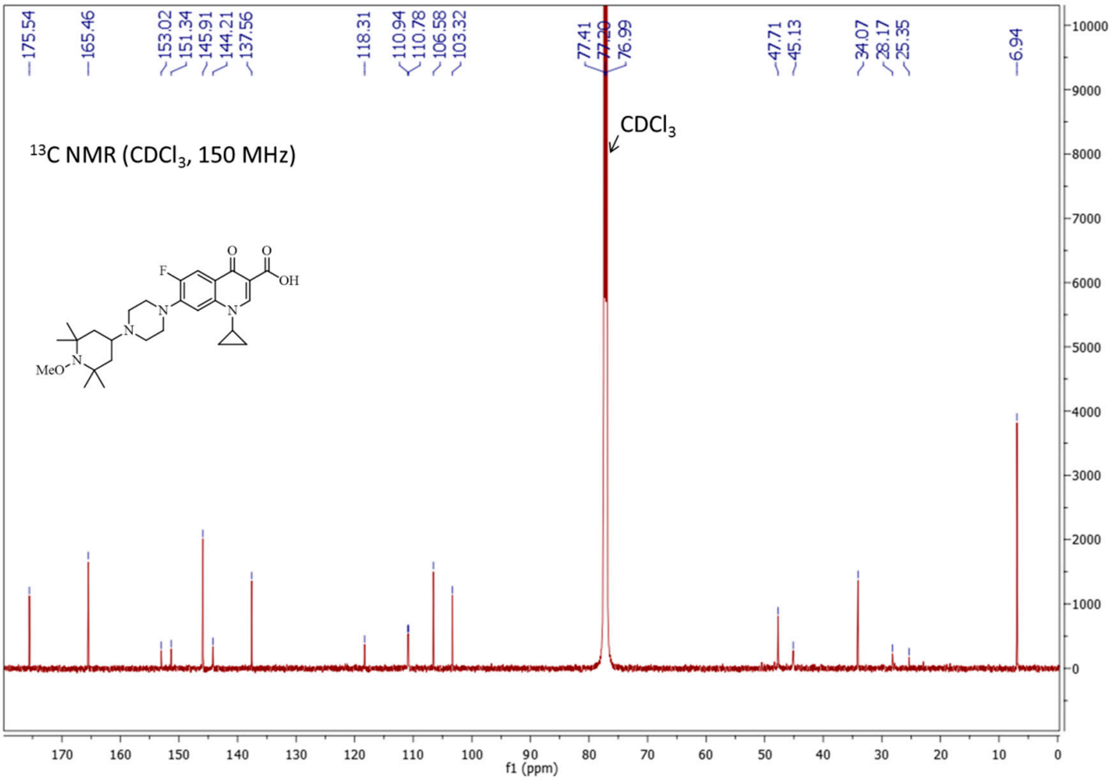

Figure S13. ^{13}C-NMR spectrum of **11**.

Column Type: Agilent Zorbax RX-SIL column (4.6 × 250 mm, 5 µm).
Flow Rate: 1 mL/min.
Solvent Composition: (DCM:THF, 70:30).

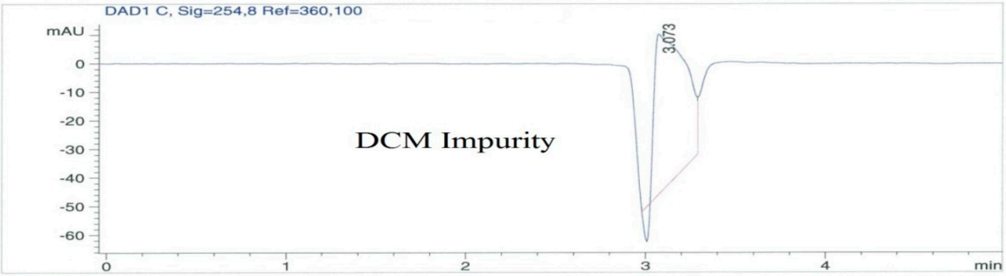

Peak No.	Retention Time (minutes)	Area	Main Pe
1	3.073	617.865	100.000

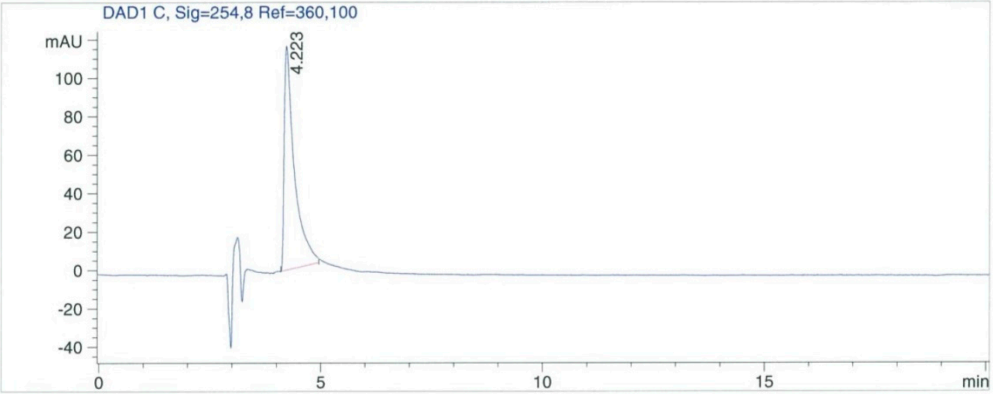

Peak No.	Retention Time (minutes)	Area	Main Pe
1	4.223	1842.200	100.000

Figure S14. HPLC chromatogram of **11**.

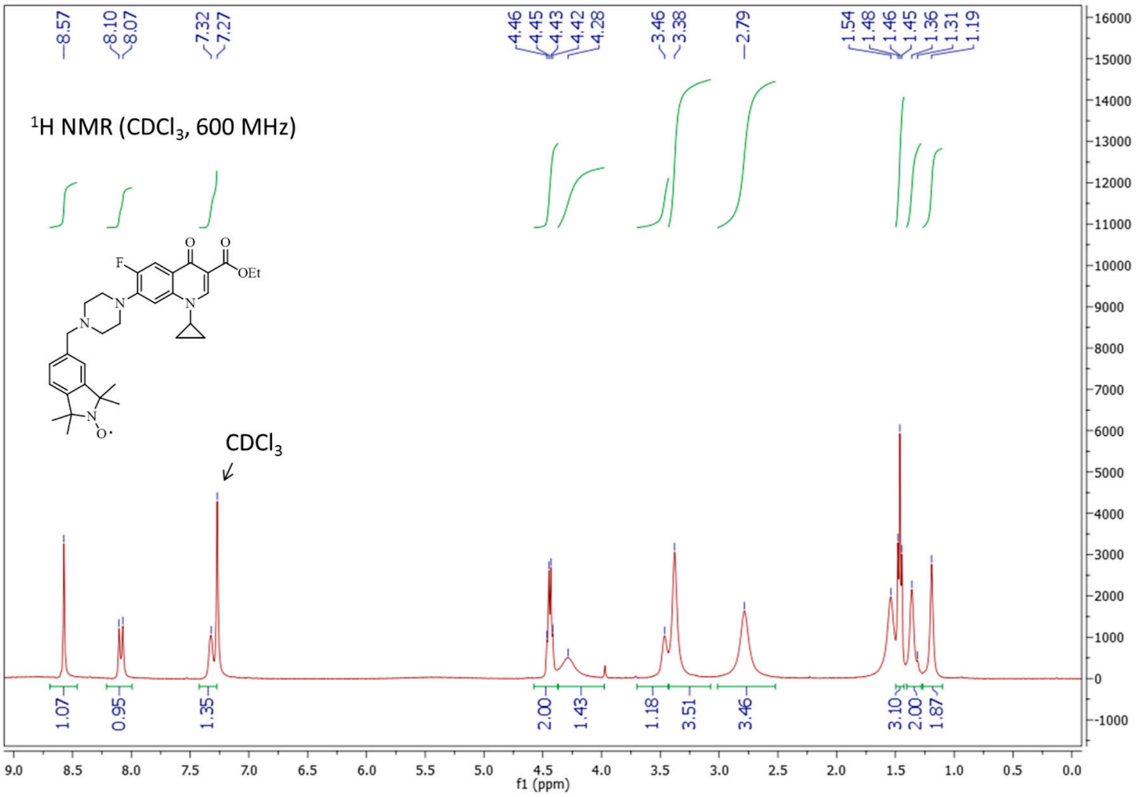

Figure S15. ¹H-NMR spectrum of **14**.

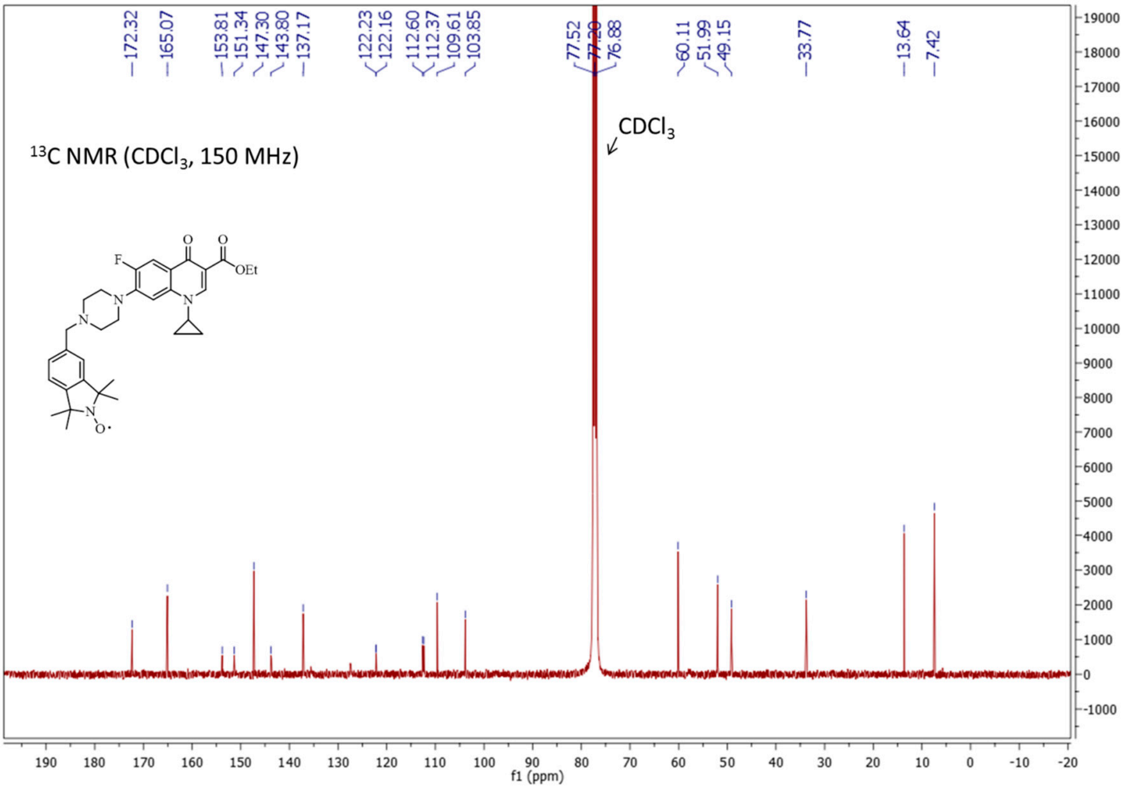

Figure S16. ^{13}C-NMR spectrum of **14**.

Column Type: Agilent C18 column (4.6 × 250 mm, 5 μm).
Flow Rate: 1 mL/min.
Solvent Composition: (MeOH:H_2O, 80:20).

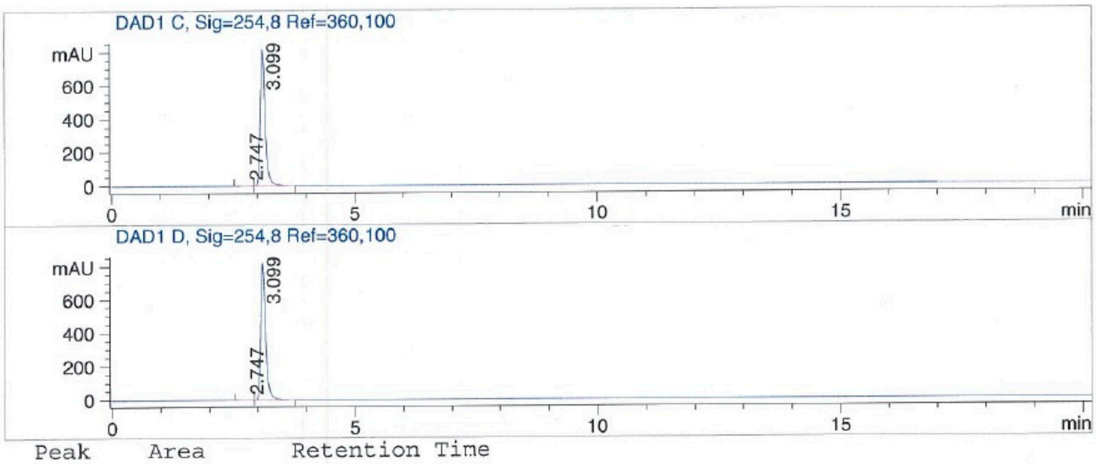

Peak No.	Area (%)	Retention Time (minutes)
1	0.274	2.747
2	99.726	3.099

Figure S17. HPLC chromatogram of **14**.

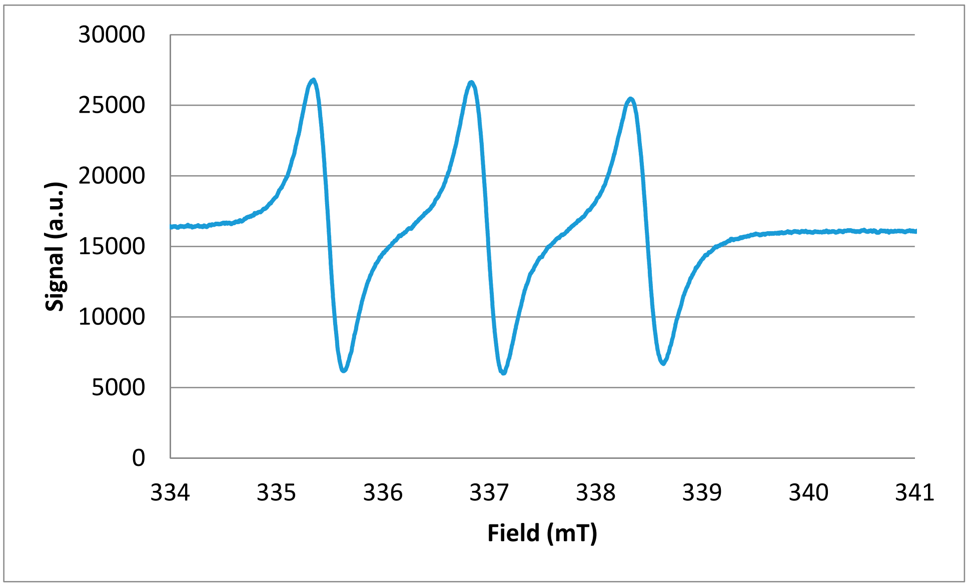

Figure S18. EPR spectrum of **14**.

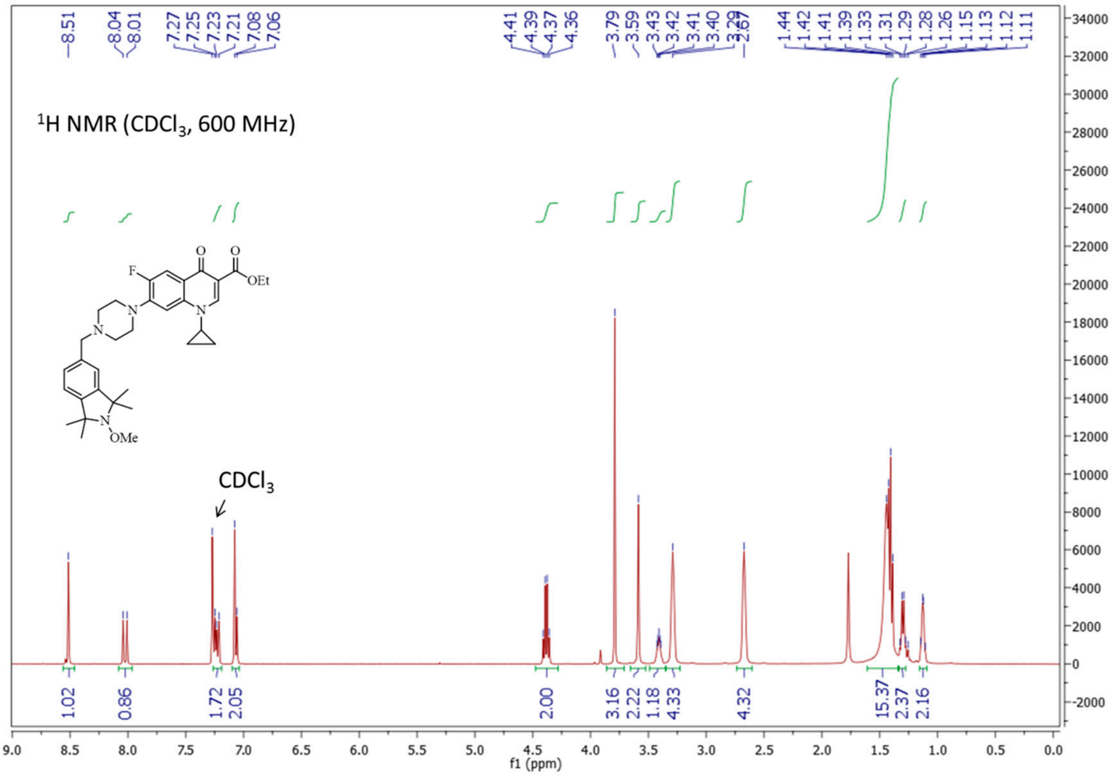

Figure S19. ^{1}H-NMR spectrum of **15**.

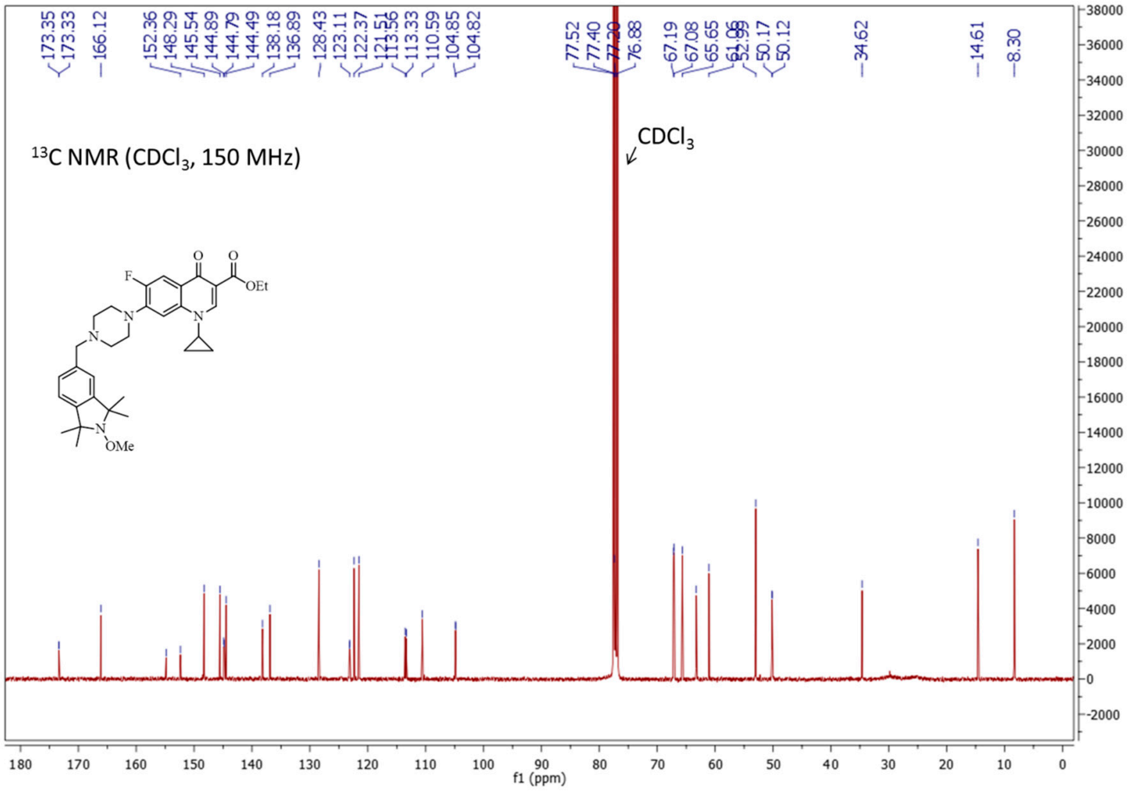

Figure S20. ^{13}C-NMR spectrum of **15**.

Column Type: Agilent C18 column (4.6 × 250 mm, 5 μm).
Flow Rate: 1 mL/min.
Solvent Composition: (MeOH:H_2O, 80:20).

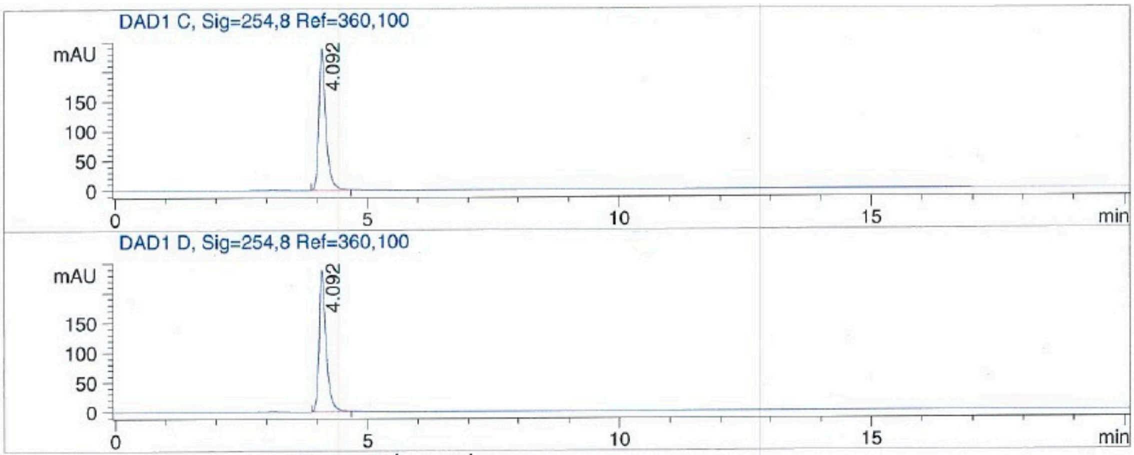

Peak No.	Area (%)	Retention Time (minutes)
1	100.000	4.092

Figure S21. HPLC chromatogram of **15**.

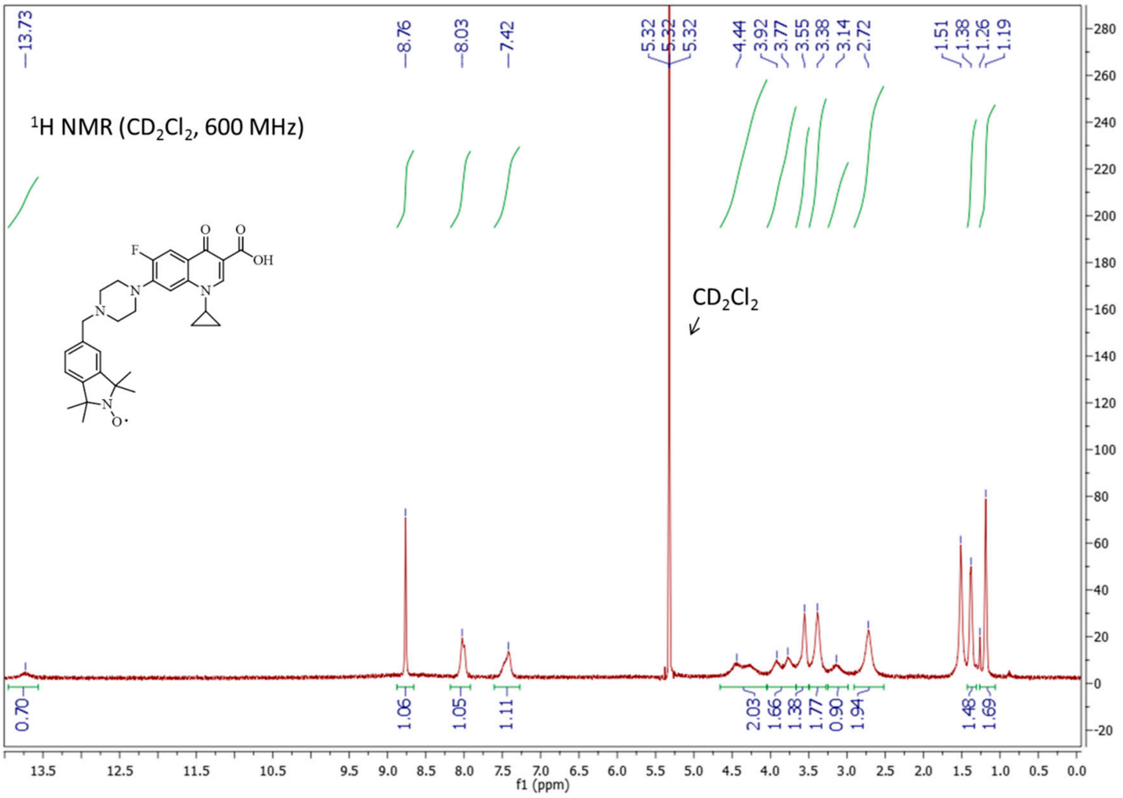

Figure S22. ¹H-NMR spectrum of **16**.

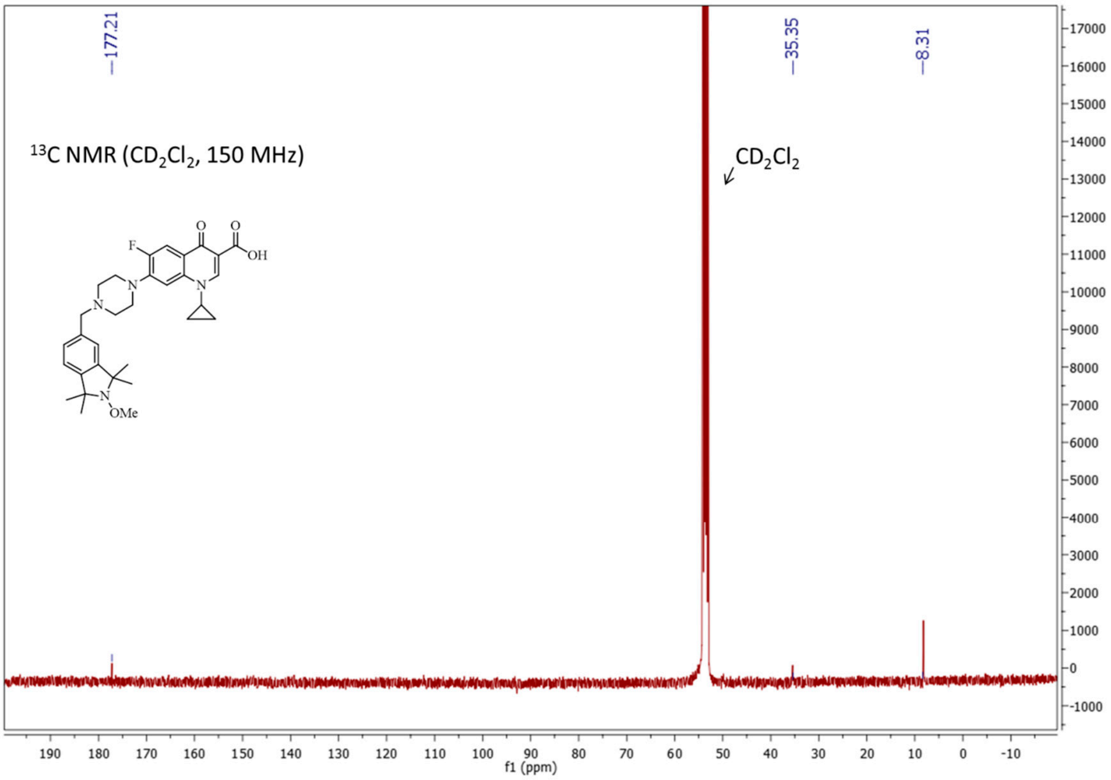

Figure S23. ¹³C-NMR spectrum of **16**.

Column Type: Agilent C18 column (4.6 × 250 mm, 5 μm).
Flow Rate: 1 mL/min.
Solvent Composition: (MeCN:H_2O/TFA(99:1), 95:5).

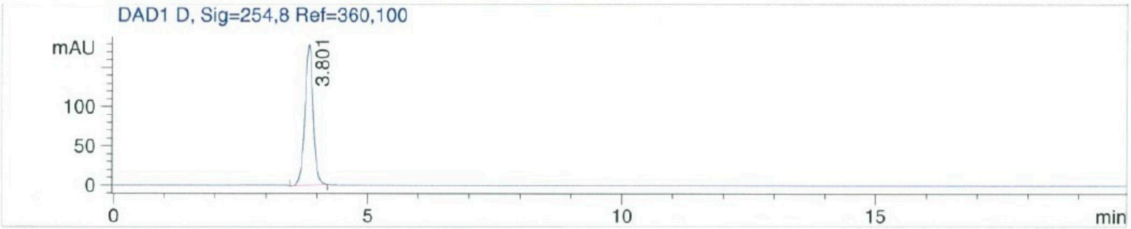

Peak No.	Area (%)	Retention Time (minutes)
1	0.000	0.000
2	0.000	0.000
3	100.000	3.801

Figure S24. HPLC chromatogram of **16**.

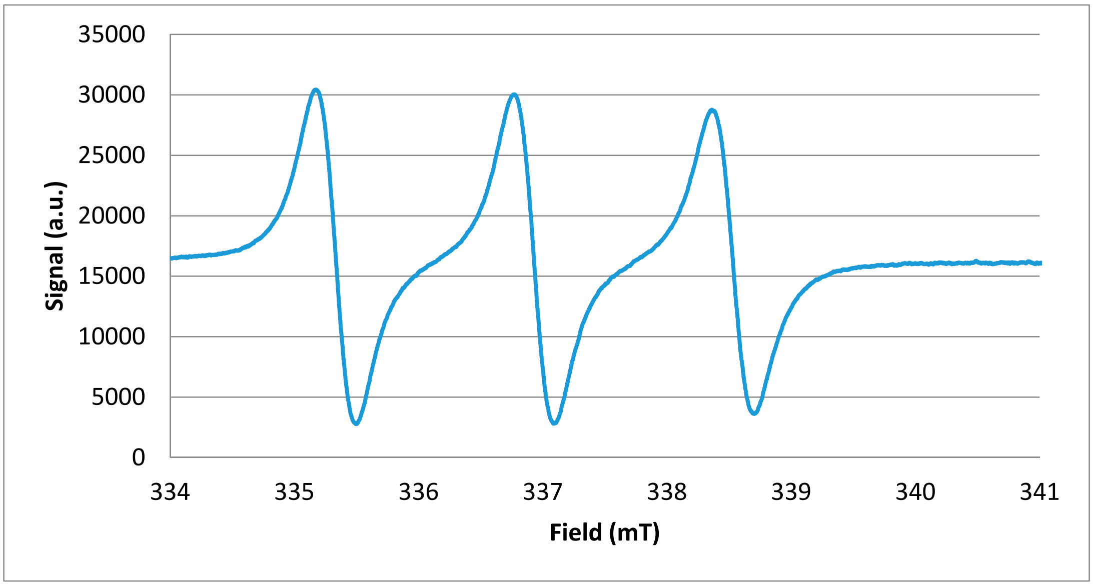

Figure S25. EPR spectrum of **16**.

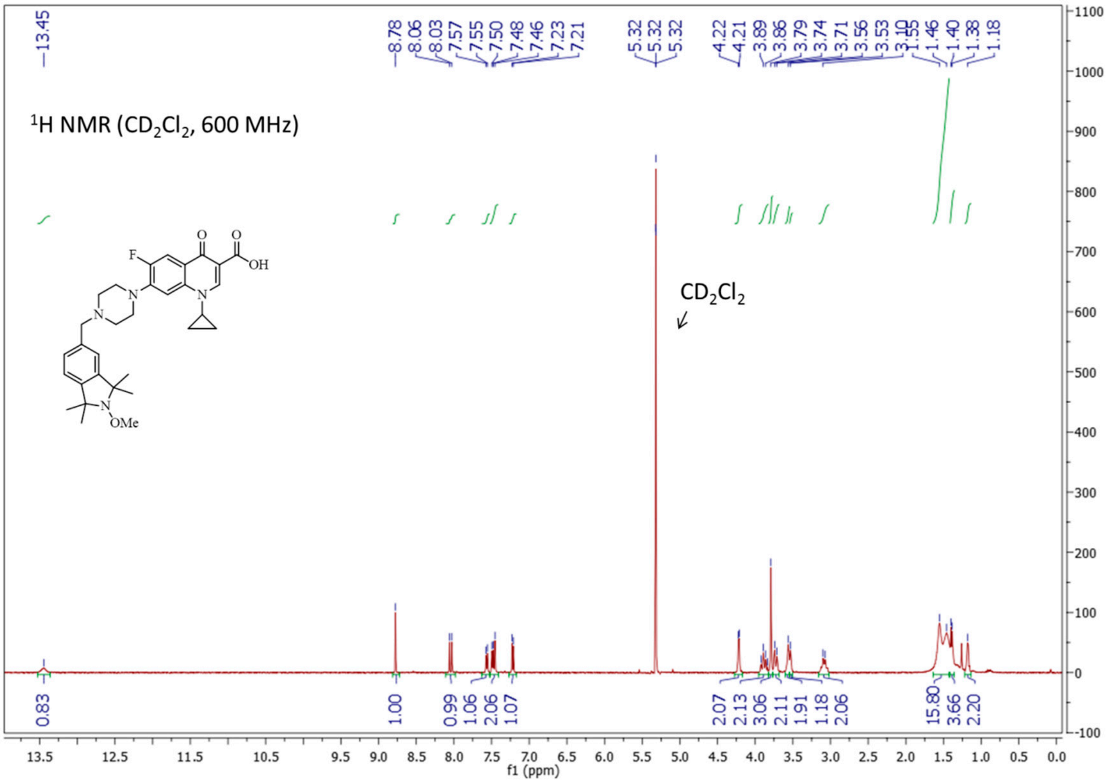

Figure S26. ^{1}H-NMR spectrum of **17**.

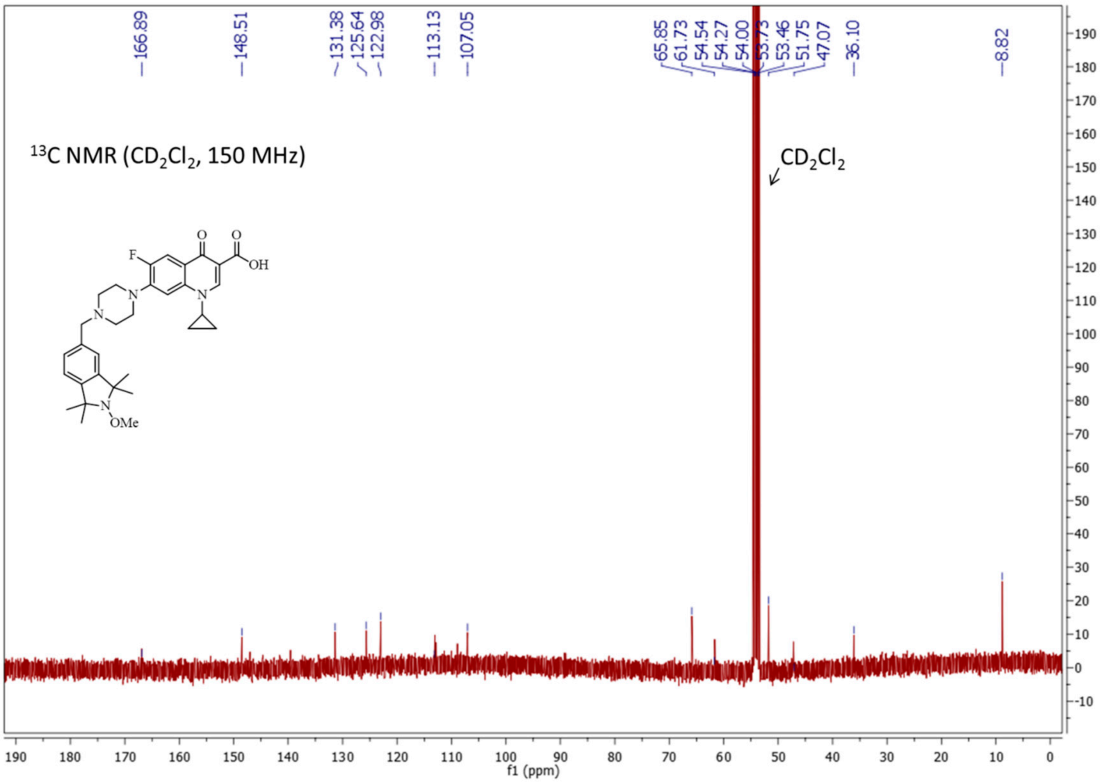

Figure S27. ^{13}C-NMR spectrum of **17**.

Column Type: Agilent C18 column (4.6 × 250 mm, 5 μm).
Flow Rate: 1 mL/min.
Solvent Composition: (MeCN:H_2O/TFA(99:1), 95:5).

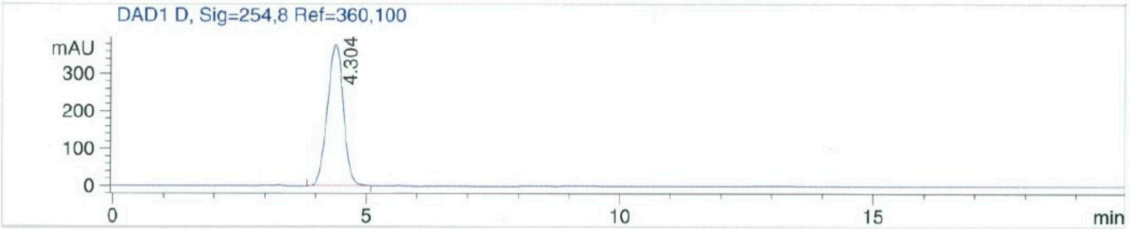

Peak No.	Area (%)	Retention Time (minutes)
1	0.000	0.000
2	100.000	4.304

Figure S28. HPLC chromatogram of **17**.
